# Differential Regulation of Intestinal Glucose Transporters SGLT1 and GLUT2 by Lactobacillaceae-Derived Heat-Killed Probiotics and Cell-Free Probiotic Supernatants

**DOI:** 10.3390/nu18172830

**Published:** 2026-08-28

**Authors:** Maša Kozmos, Tamara Hribernik, Martin Kozmos, Tomaž Langerholc, Mario Gorenjak

**Affiliations:** 1 Department of Microbiology, Biochemistry, Molecular Biology and Biotechnology, Faculty of Agriculture and Life Sciences, University of Maribor, Pivola 10, 2311 Hoče, Slovenia; tamara.hribernik2@um.si (T.H.); martin.kozmos@um.si (M.K.); tomaz.langerholc@um.si (T.L.); 2 Centre for Human Molecular Genetics and Pharmacogenomics, Faculty of Medicine, University of Maribor, Taborska ulica 8, 2000 Maribor, Slovenia; mario.gorenjak@um.si

**Keywords:** heat-killed probiotics, cell-free probiotic supernatants, glucose transporters, epithelial cell models

## Abstract

**Background/Objectives:** Although paraprobiotics and postbiotics may beneficially affect glucose metabolism, their direct influence on intestinal glucose transporters remains poorly understood. This study investigated the in vitro effects of selected heat-killed Lactobacillaceae strains and cell-free supernatants from their viable counterparts on sodium-driven glucose cotransporter 1 (SGLT1) and facilitative glucose transporter 2 (GLUT2) expression and basolateral glucose accumulation. **Methods:** Experiments were performed using non-carcinogenic porcine-derived enterocytes (CLAB) and human epithelial colorectal adenocarcinoma cells (Caco-2; ATCC HTB-37). SGLT1 and GLUT2 gene and protein expression, as well as basolateral glucose concentration after a 6-h incubation, were assessed. **Results:** Heat-killed probiotics and cell-free probiotic supernatants generally increased SGLT1 protein expression in both cell models, except for heat-killed *Lactobacillus acidophilus*. In contrast, GLUT2 modulation differed between cell models and treatments. In CLAB cells, GLUT2 protein levels were generally reduced, particularly following exposure to cell-free probiotic supernatants, whereas responses in Caco-2 cells were less consistent. Treatment-dependent changes in basolateral glucose concentration and 6-h net basolateral glucose accumulation were also observed, with significant effects for selected heat-killed strains in CLAB cells. **Conclusions:** Lactobacillaceae-derived heat-killed preparations and cell-free supernatants may differentially modulate intestinal glucose transporter expression in a strain- and cell-model-dependent manner. However, changes in transporter expression and basolateral glucose accumulation do not establish a transporter-specific glucose flux or mechanism. Further mechanistic studies using complementary intestinal models are required to clarify these effects and their translational relevance.

## 1. Introduction

Soluble carbohydrates (sugars) serve multicellular organisms as a source of carbon atoms, osmolytic molecules, and various signalling molecules, as well as a temporary source of energy storage and transport molecules. Glucose occurs in free form as a monosaccharide, but also in the form of disaccharides (maltose) or polysaccharides such as starch and glycogen [1]. In multicellular organisms, some cells (e.g., intestinal and liver cells in animals and humans) are specialised, among other things, in supplying other cells mainly with sugar. However, some cells in organisms do not have this function and are completely dependent on an external food supply (e.g., brain cells in animals and humans) [2]. The above-mentioned cellular exchange of sugars requires specific transport proteins (transporters) that support and mediate the passage (transport) of substances from cells or intercellular spaces and are therefore essential for plant, animal and human organisms. Currently, three families of solute carrier transporters (SLC) are known to be involved in glucose transport: the sodium-driven glucose transporter (SGLT) (SLC5) [3,4], the facilitative glucose transporter (GLUT) (SLC2) and the SWEET transporter (SLC50) [1]. SGLT belongs to a family of numerous membrane proteins (transporters) that play an important role in the transport of glucose, amino acids, vitamins, osmolytic molecules and some ions. Six SGLT proteins have been identified in humans and detected in different parts of the body [5,6,7]. Five SGLTs were discovered in pigs [8,9,10]. GLUTs are proteins that are encoded in the *SLC2* gene. In humans, 14 members of a large family have been identified, which are divided into three classes [11]. In pigs, 12 GLUTs were identified, but these were only detected in a few organs [8]. SGLT1 and GLUT2 are among the most important monosaccharide transporters in the intestine and are therefore responsible for glucose absorption [12].

Although the use of probiotics as modulators of the intestinal microbiota and beneficial effectors of glucose metabolism and its homeostasis has already been investigated [13,14,15,16,17,18], their effect on intestinal glucose transport in in vitro human [19,20,21,22] and animal studies [22,23,24,25,26,27] has not yet been fully explored. Recently, due to their beneficial effects on host health and potential safety even in susceptible or more sensitive individuals, non-living parts (damaged or undamaged) of probiotic microorganisms (paraprobiotics) and their metabolites (postbiotics) have attracted greater interest [28,29,30,31,32,33,34,35,36,37,38]. Moreover, a few animal studies [39,40,41,42,43,44,45,46,47,48,49,50,51,52,53,54,55,56,57] and human clinical trials [58,59,60] have described the effects of potential paraprobiotics and/or postbiotics on glucose metabolism, but none has directly focused on their effect on glucose transporters in gut epithelial cells.

Therefore, we aimed to investigate the effect of heat-killed probiotic strains of *Lactiplantibacillus* (formerly *Lactobacillus* [61]) *plantarum* PCS 20 (*L. plantarum* PCS 20), *Lactiplantibacillus* (formerly *Lactobacillus* [61]) *plantarum* PCS 26 (*L. plantarum* PCS 26), *Lacticaseibacillus* (formerly *Lactobacillus* [61]) *rhamnosus* GG (*L. rhamnosus* GG) (ATCC 53103) and *Lactobacillus acidophilus* (*L. acidophilus*) (ATCC 4356) and the cell-free supernatants of their viable strains on SGLT1 and GLUT2 gene and protein expression, as well as on glucose concentration in two intestinal epithelial cell models of human and porcine origin. The two intestinal epithelial models were selected to examine the responses in a non-carcinogenic porcine-derived enterocyte model (CLAB) and an established human enterocyte-like model (Caco-2), providing two complementary intestinal epithelial experimental systems of different origin.

## 2. Materials and Methods

### 2.1. The Growth and Preservation of Cell Cultures

Non-carcinogenic porcine-derived enterocytes (CLAB) were isolated in our laboratory [62,63,64,65] and maintained by our faculty (Faculty of Agriculture and Life Sciences, University of Maribor, Hoče, Slovenia). Due to the lack of standardised STR profiling systems for porcine intestinal epithelial cell lines, CLAB authentication is performed using species origin verification according to the PCR-based authentication assay by Gorenjak et al. [66], phenotypic characterisation (epithelial morphology) and relevant molecular markers (cytokeratins 5–18 (positive), 19 (positive), 18 (negative); actin (negative as a marker of the myofibroblast phenotype in the original phenotypic characterisation); alkaline phosphatase (positive); desmin (positive); PAS staining (positive); weakly positive for non-specific esterases, amido-black staining and acid-phosphatase; negative for markers of cells of myeloid origin). The CLAB cell line is registered in the Cellosaurus database (Accession Number: CVCL_6A78; RRID: CVCL_6A78).

Human epithelial colorectal adenocarcinoma cells (Caco-2) were directly purchased from American Type Culture Collection (ATCC (HTB-37), Manassas, VA, USA) and used in experiments within 6 months of receipt. Prior to the experiments, both cell lines were checked for possible mycoplasma infection with the Venor^®^GeM kit (Minerva Biolabs, Berlin, Germany) according to the manufacturer’s recommendations.

Both cell lines were grown in Dulbecco’s Modified Eagle’s (DMEM) advanced medium (Life Technologies, Carlsbad, CA, USA), supplemented with 100 IU/mL penicillin and 0.1 mg/mL streptomycin (both from Sigma-Aldrich, St. Louis, MO, USA), 2 mM L-glutamine and 5% foetal bovine serum (FBS) (both from Life Technologies, Carlsbad, CA, USA). Cells were incubated in 25 cm^2^ cell culture flasks (Corning Inc., Corning, NY, USA) under controlled humidified conditions (37 °C, 5% CO_2_) according to the manufacturer’s instructions. The medium was changed when necessary and the cells were passaged several times before use in the experiments (passages 28–30 for CLAB and 29–33 for Caco-2). For each analysis, three independent biological experiments were performed. Each experiment was derived from a separate cell flask.

### 2.2. The Growth and Preservation of Probiotic Strains

*Lactiplantibacillus* (formerly *Lactobacillus*) *plantarum* PCS 20 (*L. plantarum* PCS 20) and *Lactiplantibacillus* (formerly *Lactobacillus*) *plantarum* PCS 26 (*L. plantarum* PCS 26) were isolated from a Slovenian homemade cheese product [22,67,68,69,70,71,72,73]. Stock cultures of PCS 20, PCS 26, *Lacticaseibacillus* (formerly *Lactobacillus*) *rhamnosus* GG (*L. rhamnosus* GG) (ATCC 53103) and *Lactobacillus acidophilus* (*L. acidophilus*) (ATCC 4356) were kept at –80 °C in de Man, Rogosa and Sharpe (MRS) broth (Merck KGaA, Darmstadt, Germany), containing 20% (*v*/*v*) glycerol (Merck KGaA, Darmstadt, Germany). All strains were grown in MRS broth (Merck KGaA, Darmstadt, Germany) for 24 h at 35 °C in a CO_2_-enriched atmosphere according to the manufacturer’s instructions and subcultured at least three times before use in the experiments. All probiotic strains were prepared independently for each experiment.

### 2.3. Inactivation of Probiotic Strains and Preparation of Cell-Free Probiotic Supernatants

For the experiments with heat-killed probiotic strains, 10^7^ CFU/mL [68,74,75,76] of *L. plantarum* PCS 20, *L. plantarum* PCS 26, *L. rhamnosus* GG and *L. acidophilus* suspensions in MRS broth were heated to 70 °C for 30 min, 80 °C for 20 min, and 95 °C for 10 min [77,78] at 600 rpm in a thermoblock (Thermo Fisher Scientific, Waltham, MA, USA). The heat-killing process at 95 °C for 10 min [30] was used in further experiments. Subsequently, the heat-killed probiotic suspensions were cooled down to room temperature and centrifuged at 2500× *g* rpm for 10 min. MRS broth was then thoroughly discarded, and the pellet was washed twice with DMEM supplemented with 2 mM L-glutamine (both from Life Technologies, Carlsbad, CA, USA), thereby reducing the potential for residual MRS components to affect cell viability or interfere with subsequent glucose measurements. Subsequently, the pellet was resuspended in the same medium. The success of the heat-killing process was verified on MRS agar plates.

For experiments with cell-free probiotic supernatants of viable probiotic strains, *L. plantarum* PCS 20, *L. plantarum* PCS 26, *L. rhamnosus* GG and *L. acidophilus* were grown in DMEM supplemented with 2 mM L-glutamine for 6 h at a concentration of 10^7^ CFU/mL [68,74,75,76]. Afterwards, the probiotic suspensions were centrifuged at 2500× *g* rpm for 10 min and the resulting supernatants were filter-sterilised (0.2 μm). The success of the centrifugation and sterilisation process was checked on MRS agar plates, before the supernatants were added to the cells.

### 2.4. MTT-Reducing Activity Test

Both cell lines were cultured in DMEM medium (Section 2.1) at a concentration of 5 × 10^4^ cells/well in a 96-well flat-bottom microtiter plate (Corning Inc., Corning, NY, USA). The plate was incubated in a humidified atmosphere with 5% CO_2_ at 37 °C. The metabolic activity of the exposed cells was measured using the MTT (3-(4,5-dimethylthiazol-2-yl)-2, 5-diphenyltetrazolium bromide) (Sigma-Aldrich, St. Louis, MO, USA) test [79,80,81]. After 6 h of incubation (with heat-killed probiotic strains and cell-free probiotic supernatants), cells were washed twice with sterile DMEM medium (200 µL) supplemented with 2 mM L-glutamine. Subsequently, 200 µL MTT solution/well (final concentration: 0.5 mg/mL MTT) in DMEM medium supplemented with 2 mM L-glutamine was added. The plate was shaken on an orbital horizontal shaker for 5 min and then incubated in a humidified atmosphere with 5% CO_2_ at 37 °C for 1–3 h. After incubation, the medium with the added MTT solution was discarded. The plate was completely dried at room temperature. A volume of 100 µL of 0.04% HCl (Sigma-Aldrich, St. Louis, MO, USA) in isopropanol (Sigma-Aldrich, St. Louis, MO, USA) was added to each well and the plate was incubated again for 5 min on an orbital shaker. A further 15–20 min incubation at room temperature was used to dissolve the formazan crystals and subsequently generate the colour. Absorbance was measured at 570 nm and 630 nm using an M1000 PRO spectrophotometer (Tecan Trading AG, Männedorf, Switzerland). The final absorbance was calculated as the difference between the absorbance at 570 nm and 630 nm. The absorbance values of the wells with added probiotic strains were expressed as a percentage relative to the absorbance values in the control wells, which were set to 100%.

### 2.5. Gene Expression Assay

Both cell lines were cultured in DMEM medium (Section 2.1) at a concentration of 4 × 10^5^ cells/well in a 12-well flat-bottom Corning™ Costar™ microtiter plate (Corning Inc., Corning, NY, USA). The plate was incubated in a humidified atmosphere with 5% CO_2_ at 37 °C. Once confluent, the cells received fresh medium three more times. Five and seven days post-confluence for CLAB and Caco-2 cells, respectively, the plate was washed twice with warm phosphate-buffered saline (PBS) buffer (Sigma-Aldrich, St. Louis, MO, USA) to remove antibiotics. The heat-killed probiotic strains and the cell-free supernatants of viable probiotic strains were prepared as previously described (Section 2.3). One millilitre of each heat-killed probiotic suspension or cell-free supernatant was added to the cells. As a control, DMEM medium alone was used. Cells were incubated under controlled humidified conditions (37 °C, 5% CO_2_) for 6 h. A 6-h experimental window was used, as this duration was previously established as the optimal time frame for cell exposure [22,68,74,75,76]. After incubation, RNA and proteins were extracted using the NucleoSpin^®^ TriPrep isolation kit (MACHEREY-NAGEL GmbH & Co. KG, Düren, Germany) according to the manufacturer’s instructions.

The quality of extracted RNA was subsequently analysed using the Tecan NanoQuant plate and Infinite M1000 PRO microplate reader (Tecan Group Ltd., Männedorf, Switzerland). Optical density readings at 260 nm and 260/280 nm were used to determine the concentration and purity, respectively. 1 µg of extracted RNA per sample was transcribed into cDNA in a total reaction volume of 20 µL using the high-capacity cDNA reverse transcription kit (Applied Biosystems Inc., Foster City, CA, USA) according to the manufacturer’s instructions. The reverse-transcription reaction contained 10× reverse-transcription buffer, 25× dNTP mix, 10× random primers, MultiScribe™ Reverse Transcriptase, RNase inhibitor, PCR-grade RNase-free water, and 1 µg of RNA template. Reverse transcription was performed at 25 °C for 10 min, followed by 37 °C for 120 min and 85 °C for 5 min, with subsequent holding at 4 °C. No-reverse-transcription controls (no-RT), in which reverse transcriptase was omitted, were included to monitor potential genomic DNA contamination.

Prior to quantitative analysis, primer annealing conditions and amplicon specificity were experimentally evaluated using gradient PCR followed by agarose-gel electrophoresis. The target and reference genes were selected based on a review of scientific publications (Table 1). The mRNA sequences of the target and reference genes were obtained from the National Center for Biotechnology Information (NCBI) Reference Sequence (RefSeq) database and the AceView database. The corresponding RefSeq accession numbers are provided in Table 1. The primers for all target genes were designed using the online bioinformatics tool IDT OligoAnalyzer (https://eu.idtdna.com/pages/tools/oligoanalyzer accessed on 15 January 2021) and the bioinformatics tool ClustalW2 (http://www.ebi.ac.uk/Tools/, accessed on 21 July 2026) except for the *Beta-2-microglobulin (B2M)* reference gene, whose primer sequences were obtained from a previously published source [82]. All primers were supplied by Sigma-Aldrich. A volume of 2 µL of 10-fold diluted cDNA (5 ng/µL) with sterile PCR-grade RNase-free water was then used for quantitative real-time PCR (qPCR) gene expression analysis. qPCR was performed using 2× Maxima SYBR Green qPCR Master Mix (Thermo Fisher Scientific, Waltham, MA, USA) on a LightCycler^®^ 96 Real-Time PCR System (Roche, Basel, Switzerland). Each 10-µL reaction contained 5 µL of 2× Maxima Master Mix, 1.2 µL of each primer (2.5 µM stock concentration; 0.3 µM final concentration), 0.6 µL of PCR-grade water, and 2 µL of diluted cDNA. No-template controls (NTCs), in which the cDNA template was replaced with PCR-grade water, were included to monitor potential reagent contamination and nonspecific amplification. Amplification was performed with an initial denaturation at 95 °C for 10 min, followed by 40 cycles of denaturation at 95 °C for 15 s, annealing at 60 °C for 30 s, and extension at 72 °C for 30 s. Following amplification, melting-curve analysis was performed to assess amplicon specificity. Primer-pair amplification performance was evaluated before quantitative analyses using standard curves generated for each primer pair to verify comparable amplification efficiencies. The original numerical amplification-efficiency, slope, and R^2^ values from these standard curves are no longer available from the archived experimental dataset and were therefore not retrospectively estimated.

Ct values generated by the LightCycler^®^ 96 software were exported and processed using predefined Excel calculation templates. For each biological replicate, target-gene Ct values were normalised to the geometric mean of *RNA* reference genes *B2M* and *Polymerase II Subunit A (POLR2A)* for CLAB cells and *Actin Beta (ACTB)* and *POLR2A* for Caco-2 cells to obtain ΔCt values. Treatment-specific ΔCt values were then compared with the corresponding control to calculate ΔΔCt, and relative gene expression was expressed as 2^−ΔΔCt^ [83]. Stability of the selected reference genes under the experimental conditions was evaluated before target-gene analysis. Three independent biological replicates were analysed for each experimental condition; no additional technical replicates were performed. The exact historical LightCycler^®^ 96 Ct-threshold/quantification-algorithm settings are no longer available and therefore could not be retrospectively verified.

### 2.6. SGLT1 and GLUT2 Protein Quantification

After extraction using the NucleoSpin^®^ TriPrep isolation kit (MACHEREY-NAGEL GmbH & Co. KG, Düren, Germany), protein supernatants were used directly for protein concentration measurements or stored at −20 °C until further use. Bradford reagent (Sigma-Aldrich, St. Louis, MO, USA) was used to measure protein concentration in the supernatants at 570 nm according to the manufacturer’s instructions (NucleoSpin^®^ TriPrep isolation kit; MACHEREY-NAGEL GmbH & Co. KG, Düren, Germany) and normalised to 10 μg. Samples were mixed and incubated at 65 °C for 12 min. Afterwards, samples were centrifuged at 11,000× *g* for 1 min and the supernatants, together with the molecular-weight standard (mPAGE^®^ Western Protein Standard; Merck KGaA, Darmstadt, Germany), were separated by SDS polyacrylamide gel electrophoresis (SDS-PAGE) using mPAGE^®^ 10% Bis-Tris Precast Gel (Merck KGaA, Darmstadt, Germany). Proteins were then transferred onto Immobilon^®^-E PVDF Transfer Membrane (Merck KGaA) using the mPAGE^®^ Mini Wet Transfer System (Merck KGaA, Darmstadt, Germany). For membrane blocking and primary/secondary antibody dilutions, Immobilon^®^ Block—CH (Merck KGaA, Darmstadt, Germany) Blocker was used. The membrane was then stained for SGLT1 and GLUT2 proteins using human/pig-reactive rabbit antibody (07-1417; Merck KGaA, Darmstadt, Germany) and human/pig-reactive rabbit antibody (SAB2108554; Sigma-Aldrich, St. Louis, MO, USA), respectively. Anti-ACTIN antibody (A2066; Merck KGaA, Darmstadt, Germany) was used as a reference control. The peroxidase-conjugated secondary antibody Goat Anti-Rabbit IgG (AP132P) was purchased from Merck KGaA, Darmstadt, Germany. Blocking and staining procedures were performed using the SNAP i.d.^®^ 2.0 Protein Detection System (Merck KGaA, Darmstadt, Germany). Afterwards, samples were stained with Chemiluminescence Immobilon^®^ Crescendo Western HRP Substrate (Merck KGaA, Darmstadt, Germany), and the signal was detected by the Vilber Fusion FX6 Edge system (Vilber Lourmat SAS, Collégien, France). Images obtained at different exposure times were evaluated to select appropriate target-protein signals for densitometric analysis. The immunoreactive bands at approximately 72 kDa for SGLT1, 58 kDa for GLUT2 and 42 kDa for ACTIN were quantified based on their expected molecular weights and comparison with preliminary molecular-weight-standard-containing immunoblots (Figures S1 and S2). No additional antibody-specificity validation using positive tissue, knockdown, or peptide-competition controls was performed. Densitometric analysis was performed using Fusion FX6 Edge imaging software (version 18.08. SN; Vilber Lourmat SAS, Collégien, France), and normalisation to the intensities of reference bands was performed. Normalised intensities were expressed as a percentage relative to normalised control intensities, which were set to 100%. All directly compared samples within each experimental set were processed on the same gel and membrane under standardised electrophoresis, transfer, immunodetection, and imaging conditions.

### 2.7. Glucose Assay

Glucose concentration was evaluated using polarized enterocytes cultured on microporous membranes. CLAB and Caco-2 cells were seeded at a density of 3 × 10^5^ cells/well onto polyester and polycarbonate membrane inserts, respectively, featuring a pore size of 0.4 μm and a surface area of 1 cm^2^ (Corning Inc., Corning, NY, USA). Both cell lines were maintained in Advanced DMEM medium as previously described in Section 2.1. The plates were incubated under humidified, controlled conditions (5% CO_2_ at 37 °C), and the medium was replaced as required until both the transepithelial electrical resistance characteristic for each cell line (>500 Ω·cm^2^ for CLAB and >1000 Ω·cm^2^ for Caco-2) and a confluent, differentiated cell model (14–16 days and 19–23 days after seeding on inserts for CLAB and Caco-2, respectively) were achieved. The heat-killed probiotic strains and the cell-free probiotic supernatants were prepared as previously described (Section 2.3).

To eliminate residual antibiotics, the apical and basolateral compartments were rinsed twice with pre-warmed PBS buffer prior to any treatment. Subsequently, 0.5 mL of each suspension (heat-killed probiotic strains and cell-free probiotic supernatants), prepared in low-glucose DMEM (5.5 mM) without phenol red (Life Technologies, Carlsbad, CA, USA) and without antibiotics, supplemented with 2 mM L-glutamine (Life Technologies, Carlsbad, CA, USA) and enriched with 10 mM D-glucose (Sigma-Aldrich, Carlsbad, CA, USA), was added to the apical compartment of each cell line. As a result, the nominal initial apical glucose concentration in all inserts was 15.5 mM. The corresponding medium lacking heat-killed probiotic strains and cell-free probiotic supernatants served as the control.

In all basolateral compartments, 1.5 mL of low-glucose DMEM without phenol red (Life Technologies), supplemented with 2 mM L-glutamine (Life Technologies, Carlsbad, CA, USA), was added. Plates were then incubated for 6 h under controlled humidified conditions (5% CO_2_ at 37 °C). Following incubation, transepithelial electrical resistance was measured again to exclude any possible barrier disruption. Both cell lines maintained the transepithelial electrical resistance values recorded before treatment. Subsequently, basolateral media from each well were carefully collected, and 500 μL of the mixed basolateral medium was loaded into 10 kDa cutoff ultrafiltration columns (Abcam PLC, Cambridge, UK) and centrifuged at 10,000× g for 10 min at 4 °C to achieve sample deproteinization. After centrifugation, glucose levels in the ultrafiltrate were quantified using an enzyme-based Glucose Assay Kit (Abcam PLC, Cambridge, UK) according to the manufacturer’s protocol.

### 2.8. Statistics

The results were statistically processed using IBM SPSS Statistics 30.0 (IBM Inc., Armonk, NY, USA). Distributional assumptions were assessed using graphical inspection and the Shapiro–Wilk test, while variance homogeneity was evaluated using Levene’s test. Because normality tests have limited power at very small sample sizes, their results were treated only as supportive data diagnostics and not as proof of normality. Depending on the observed distribution and variance homogeneity, ordinary one-way ANOVA, Welch’s ANOVA, or the Kruskal–Wallis H test was applied.

For the cytotoxicity assay, gene and protein expression analyses, and glucose assay, ordinary one-way ANOVA was followed by Dunnett’s post hoc test when the assumptions of parametric analysis and homogeneity of variances were met. When variances were unequal, Welch’s ANOVA followed by Dunnett’s T3 post hoc test was applied. Data that did not meet the assumptions for parametric analysis were analysed using the Kruskal–Wallis H test. Gene expression data were statistically analysed using 2^−ΔΔCt^ values [83]. For reference gene stability, 2^−ΔCt^ values [83] from the raw data were analysed using ordinary one-way ANOVA or Welch’s ANOVA, as appropriate based on Levene’s test for homogeneity of variances.

Associations between basolateral glucose concentration and SGLT1 or GLUT2 protein expression were evaluated separately for each cell line and treatment preparation using paired treatment-level biological observations. Each analysis included 15 paired observations (*n* = 3 per treatment) obtained from three independent biological experiments. Pearson’s product-moment correlation coefficient was used when normality assumptions were met, whereas Spearman’s rank correlation coefficient was used for non-normally distributed data, as assessed using the Shapiro–Wilk test. For Pearson’s r, 95% confidence intervals were calculated using Fisher’s z transformation; the 95% confidence interval for Spearman’s ρ was estimated using bootstrap resampling. A significance level of α = 0.05 was applied to all statistical analyses, with *p* < 0.05 considered statistically significant.

## 3. Results

### 3.1. Metabolic Activity of the Cell Lines After Exposure to the Heat-Killed Probiotic Strains and the Cell-Free Probiotic Supernatants

**Figure 1:**
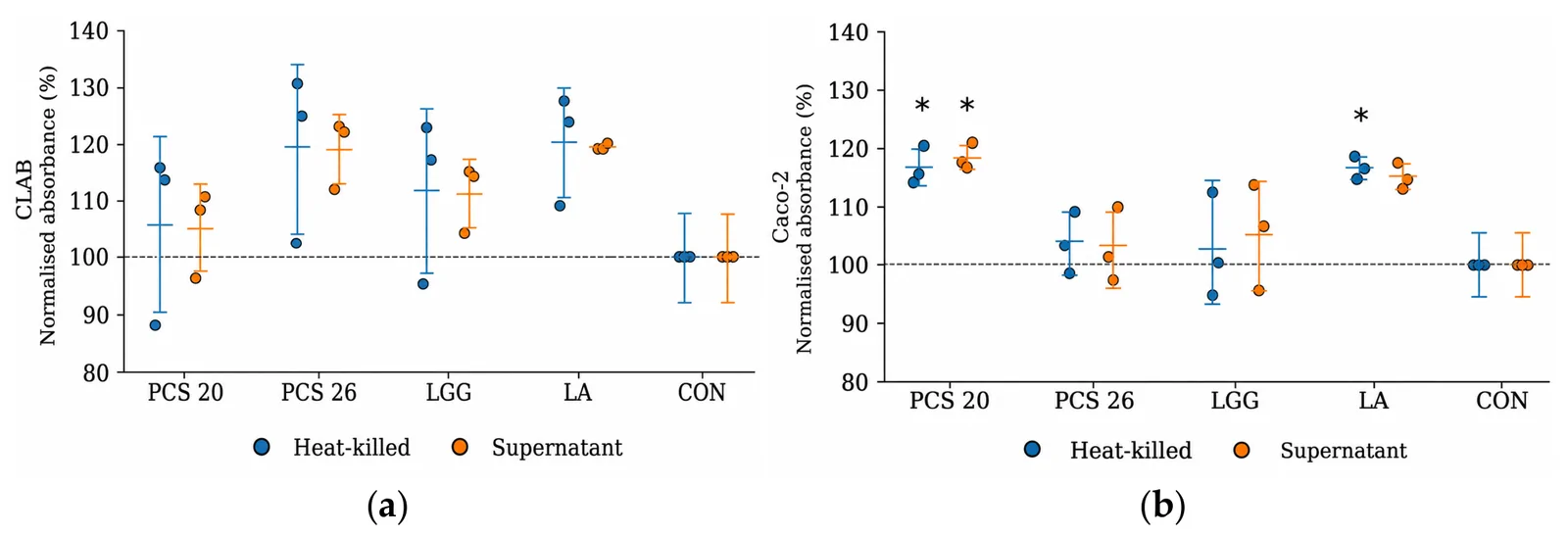
Metabolic activity test in CLAB (**a**) and Caco-2 (**b**) cells. The results show the metabolic activity measured after 6 h of incubation of cells with heat-killed probiotic strains and the cell-free supernatants of the probiotic strains *Lactiplantibacillus plantarum* PCS 20 (PCS 20), *Lactiplantibacillus plantarum* PCS 26 (PCS 26), *Lacticaseibacillus rhamnosus* GG (LGG) and *Lactobacillus acidophilus* (LA). Data are presented as mean values of normalised absorbance from three independent biological experiments (*n* = 3) ± SD. Statistical differences compared with the corresponding control (CON) were determined using one-way ANOVA followed by Dunnett’s post hoc test (* *p* < 0.05).

Before testing the effects of the heat-killed probiotic strains and the cell-free probiotic supernatants on the CLAB and Caco-2 cell lines, we assessed the metabolic activity of both cell lines after 6 h of exposure to the cell-free probiotic supernatants and heat-killed forms of *L. plantarum* PCS 20, *L. plantarum* PCS 26, *L. rhamnosus* GG, and *L. acidophilus*. All treatments resulted in increased metabolic activity in both cell lines (Figure 1a for CLAB cells and Figure 1b for Caco-2 cells).

In CLAB cells (Figure 1a), the addition of all heat-killed probiotic strains and their cell-free probiotic supernatants increased the metabolic activity of CLAB cells, but these differences were not statistically significant (*L. rhamnosus* GG by 11% and 11%, *L. plantarum* PCS 26 by 19% and 18%, *L. plantarum* PCS 20 by 6% and 5%, *L. acidophilus* by 19% and 19%, respectively).

Caco-2 cells (Figure 1b) also showed increased metabolic activity after incubation with heat-killed probiotic strains and cell-free supernatants of all probiotic strains compared to the control. Moreover, *L. plantarum* PCS 20 (17%; *p* = 0.024 and 18%; *p* = 0.005, respectively) showed statistically significant increases in both co-incubations, whereas the *L. acidophilus* supernatant significantly increased metabolic activity by 8% (*p* = 0.017). The heat-killed probiotic strains and cell-free supernatants of *L. plantarum* PCS 26 (by 3% and 3%, respectively) and *L. rhamnosus* GG (by 2% and 4%, respectively) also increased metabolic activity, but these differences were not statistically significant compared to the control.

### 3.2. Stability of Reference Gene Expression in CLAB and Caco-2 Cell Lines

To ensure the reliability of the qPCR analysis, we assessed the stability of reference gene expression in CLAB (Figure S3a) and Caco-2 (Figure S3b) cells exposed to heat-killed probiotic strains and cell-free supernatants of the probiotic strains *L. plantarum* PCS 20, *L. plantarum* PCS 26, *L. rhamnosus* GG, *L. acidophilus*, as well as medium alone (controls). There was no statistically significant difference among the samples. Therefore, the transcription of *B2M/POLR2A* genes in CLAB cells (Figure S3a) and *ACTB/POLR2A* genes in Caco-2 cells (Figure S3b) was stable (CLAB: *p* = 0.549 and *p* = 0.112; Caco-2: *p* = 0.234 and *p* = 0.059 for heat-killed probiotic strains and cell-free probiotic supernatants, respectively).

### 3.3. Relative SGLT1 and GLUT2 Gene Expression in CLAB and Caco-2 Epithelial Cells

After confirming the stability of reference gene expression, the effects of heat-killed probiotic strains and cell-free supernatants from probiotic strains *L. plantarum* PCS 26, *L. plantarum* PCS 20, *L. rhamnosus* GG and *L. acidophilus* on the expression of glucose transporter genes in CLAB and Caco-2 epithelial cells were analysed.

- *SGLT1*

**Figure 2:**
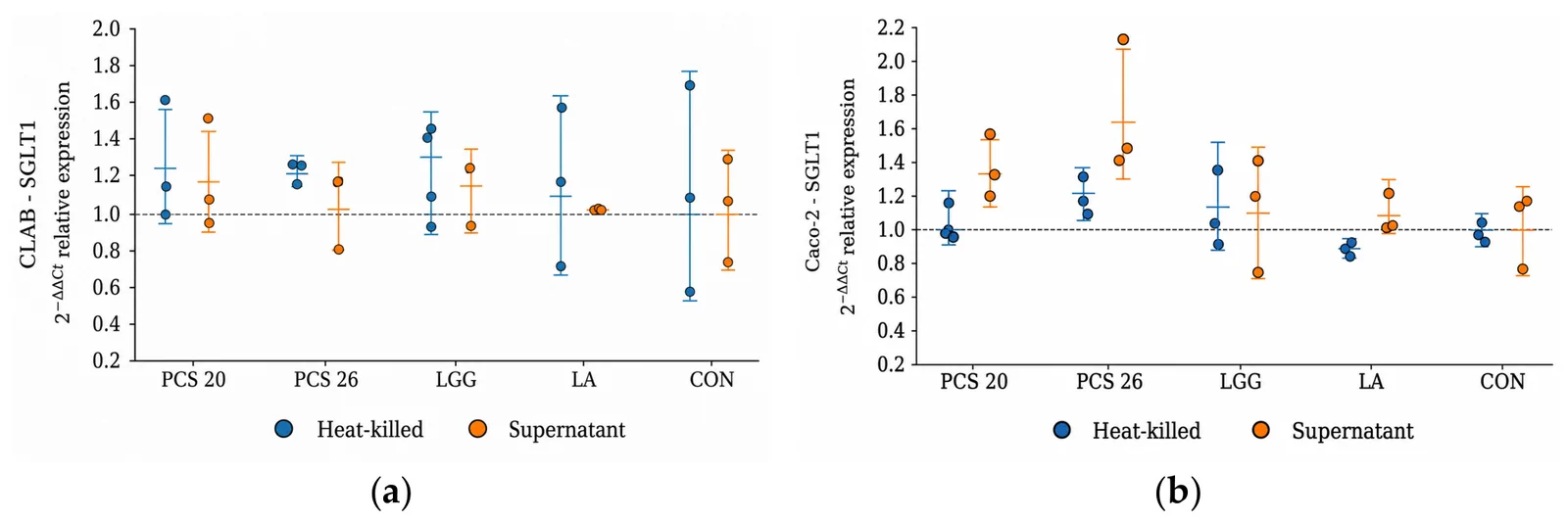
*SGLT1* relative expression in CLAB (**a**) and Caco-2 (**b**) cells. The results show the expression of the *SGLT1* gene after 6 h of incubation of cells with heat-killed probiotic strains and cell-free supernatants of the probiotic strains *Lactiplantibacillus plantarum* PCS 20 (PCS 20), *Lactiplantibacillus plantarum* PCS 26 (PCS 26), *Lacticaseibacillus rhamnosus* GG (LGG) and *Lactobacillus acidophilus* (LA). Data are presented as 2^−ΔΔCt^ from three independent biological experiments (*n* = 3) with minimal and maximal expression error. Statistical differences compared with the corresponding control (CON) were determined using ANOVA and Kruskal–Wallis H test (*p* < 0.05). SGLT1: sodium-driven glucose transporter 1.

Relative expression of the *SGLT1* gene in the CLAB cell line after co-incubation with heat-killed probiotic strains and with the cell-free supernatant of the probiotic strains is shown in Figure 2a. Compared to the control, all tested heat-killed strains (*L. plantarum* PCS 20, *L. plantarum* PCS 26, *L. rhamnosus* GG, and *L. acidophilus*) increased *SGLT1* gene expression (1.22-, 1.23-, 1.29-, and 1.09-fold, respectively), although these increases were not statistically significant. Similarly, co-incubation with the cell-free probiotic supernatants increased *SGLT1* gene expression in all samples, although the differences were not statistically significant (*L. plantarum* PCS 20—1.15-fold; *L. plantarum* PCS 26—1.02-fold; *L. rhamnosus* GG—1.11-fold; and *L. acidophilus*—1.01-fold) compared to the control.

Figure 2b shows the relative expression of the *SGLT1* gene in the Caco-2 cell line after co-incubation with heat-killed probiotic strains and with the cell-free supernatant of the probiotic strains. Compared to the control, *SGLT1* gene expression was upregulated after the addition of heat-killed *L. plantarum* PCS 20, *L. plantarum* PCS 26, and *L. rhamnosus* GG (1.03-, 1.15-, and 1.09-fold, respectively), while heat-killed *L. acidophilus* had the opposite effect, downregulating the gene 1.11-fold. None of these effects were statistically significant. Furthermore, co-incubation with the cell-free probiotic supernatants increased *SGLT1* gene expression in all samples (*L. plantarum* PCS 20—1.30-fold; *L. plantarum* PCS 26—1.60-fold; *L. rhamnosus* GG—1.06-fold; and *L. acidophilus*—1.10-fold) compared to the control. The cell-free supernatant of *L. plantarum* PCS 26 had the greatest effect; however, none of the effects were statistically significant.

- *GLUT2*

**Figure 3:**
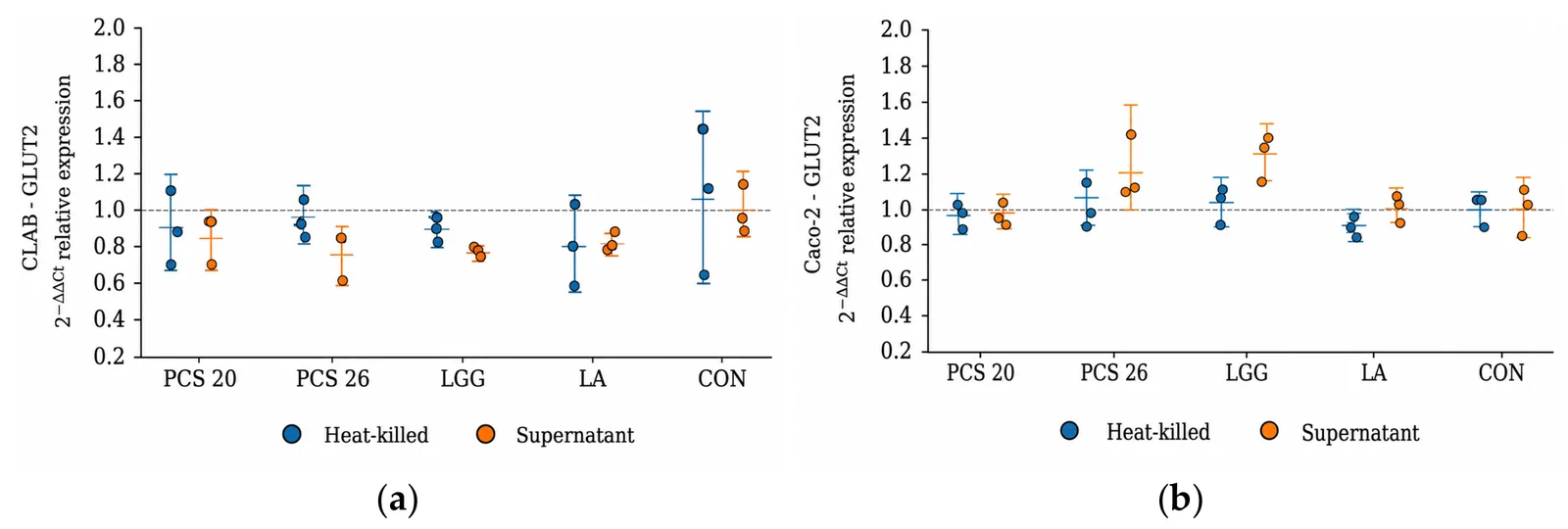
*GLUT2* relative expression in CLAB (**a**) and Caco-2 (**b**) cells. The results show the expression of the *GLUT2* gene after 6 h of incubation of cells with heat-killed probiotic strains and cell-free supernatants of the probiotic strains *Lactiplantibacillus plantarum* PCS 20 (PCS 20), *Lactiplantibacillus plantarum* PCS 26 (PCS 26), *Lacticaseibacillus rhamnosus* GG (LGG) and *Lactobacillus acidophilus* (LA). Data are presented as 2^−ΔΔCt^ from three independent biological experiments (*n* = 3) with minimal and maximal expression error. Statistical differences compared with the corresponding control (CON) were determined using ANOVA and Kruskal–Wallis H test (*p* < 0.05). GLUT2: facilitative glucose transporter 2.

Relative expression of the *GLUT2* gene in the CLAB cell line after co-incubation with heat-killed probiotic strains and with the cell-free supernatants of the probiotic strains is shown in Figure 3a. Heat-killed strains *L. plantarum* PCS 20, *L. plantarum* PCS 26, *L. rhamnosus* GG, and *L. acidophilus* tended to downregulate GLUT2 gene expression (1.10-, 1.04-, 1.09-, and 1.22-fold, respectively) compared to the control; however, the effect was not significant. Similarly, the cell-free supernatant of all probiotic strains did not significantly affect *GLUT2* gene downregulation (*L. plantarum* PCS 20—1.16-fold; *L. plantarum* PCS 26—1.24-fold; *L. rhamnosus* GG—1.23-fold; and *L. acidophilus*—1.18-fold) compared to the control.

In Caco-2 cells (Figure 3b), heat-killed *L. plantarum* PCS 20 and *L. acidophilus* had a minor, nonsignificant effect on *GLUT2* downregulation (1.03- and 1.08-fold, respectively). However, heat-killed *L. plantarum* PCS 26 and *L. rhamnosus* GG upregulated the same gene (1.05- and 1.01-fold, respectively) compared to the control, though this effect was also statistically nonsignificant. *GLUT2* gene expression in Caco-2 cells increased after the addition of *L. plantarum* PCS 26 (1.19-fold), *L. rhamnosus* GG (1.24-fold), and *L. acidophilus* (1.01-fold) cell-free supernatants compared to the control. *L. rhamnosus* GG appeared to upregulate the *GLUT2* gene the most (1.24-fold), although this was not significant. In contrast, the addition of *L. plantarum* PCS 20 cell-free supernatant downregulated *GLUT2* gene expression (1.02-fold), but the effect was minimal and not significant.

### 3.4. SGLT1 and GLUT2 Protein Expression in CLAB and Caco-2 Epithelial Cells

To assess whether probiotic-induced changes in gene transcription are also reflected in protein expression and thus determine the actual effect of probiotic strains (heat-killed and cell-free probiotic supernatants), protein samples were immunoblotted for SGLT1 and GLUT2.

- SGLT1

**Figure 4:**
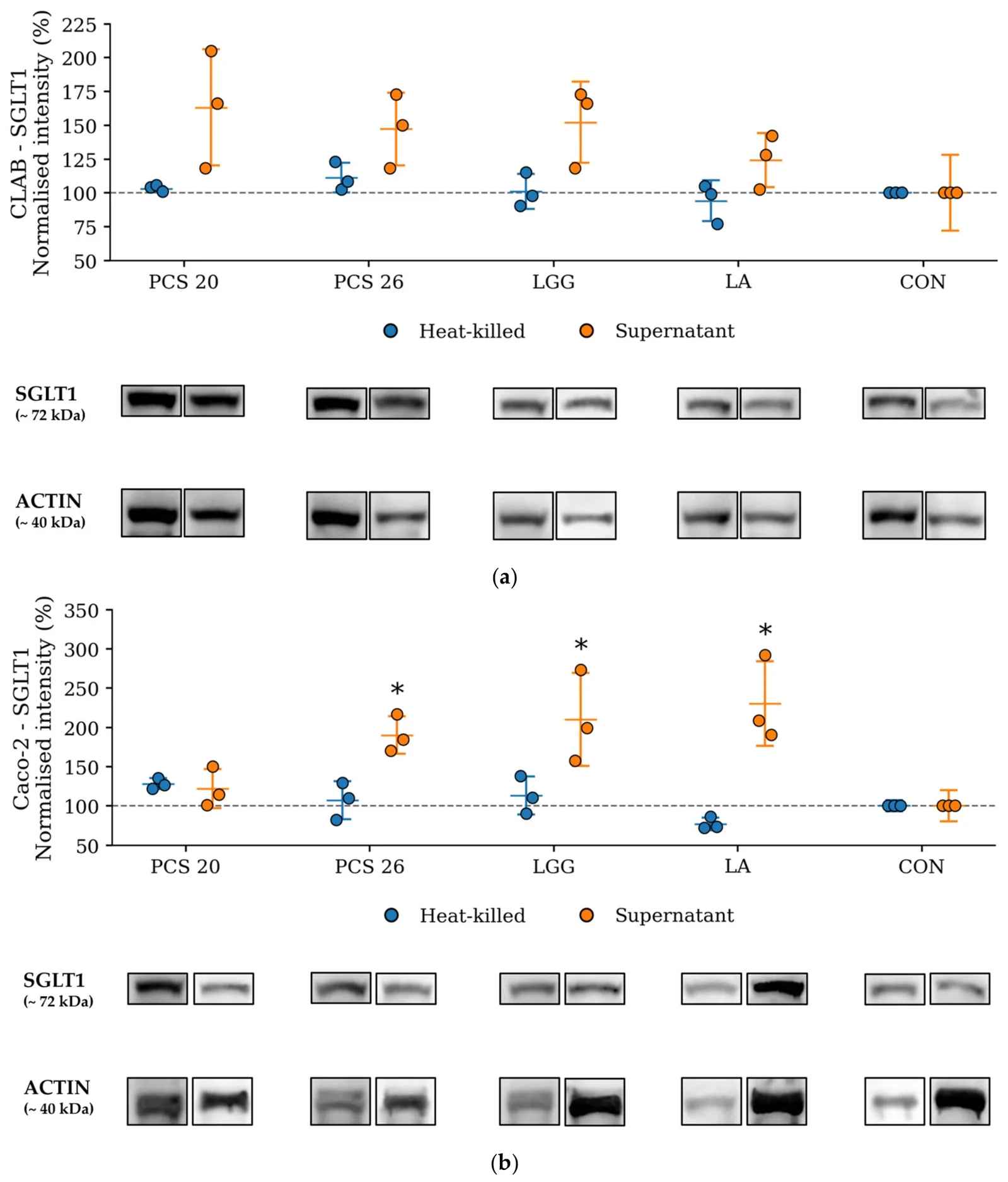
SGLT1 protein expression in CLAB (**a**) and Caco-2 (**b**) cells after 6 h of incubation with heat-killed probiotic strains and cell-free supernatants of the probiotic strains *Lactiplantibacillus plantarum* PCS 20 (PCS 20), *Lactiplantibacillus plantarum* PCS 26 (PCS 26), *Lacticaseibacillus rhamnosus* GG (LGG), and *Lactobacillus acidophilus* (LA). SGLT1 band intensities were normalised to the reference protein ACTIN and expressed as percentages relative to the corresponding control (CON), which was set to 100%. Data are presented as mean ± SD from three independent biological experiments (*n* = 3). Statistical differences compared with the corresponding control were determined using one-way ANOVA followed by Dunnett’s post hoc test (* *p* < 0.05). Representative immunoblot bands are shown for each experimental condition. Vertical lines indicate the boundaries between non-adjacent lanes originating from two separate immunoblots corresponding to the heat-killed probiotic and cell-free supernatant experiments. SGLT1 and ACTIN were detected on the same membrane. SGLT1: sodium-driven glucose transporter 1.

Heat-killed probiotic strains (*L. plantarum* PCS 20, *L. plantarum* PCS 26 and *L. rhamnosus* GG) slightly increased the SGLT1 protein levels (103%, 111%, and 101%, respectively) in CLAB cells (Figure 4a and Figure S4), consistent with the gene expression results (Figure 2a). However, the increase in SGLT1 gene transcript after heat-killed *L. acidophilus* addition was not consistent with the protein level results, where a minimal reduction in SGLT1 (94%) was detected. Furthermore, the SGLT1 protein results of all cell-free probiotic supernatants (Figure 4a and Figure S5) were consistent with the *SGLT1* gene expression results, showing an increase in protein level for *L. plantarum* PCS 20, *L. plantarum* PCS 26, and *L. rhamnosus* GG (163%, 147%, and 151%, respectively). The effect of *L. acidophilus* cell-free supernatant on protein expression was the smallest (124%), but also consistent with the gene results. However, none of these effects were statistically significant.

The effect of heat-killed probiotic strains on SGLT1 protein levels (Figure 4b and Figure S6) reflected the changes in *SGLT1* gene expression (Figure 2b) in the Caco-2 cell line. An increase in SGLT1 protein levels was observed for heat-killed *L. plantarum* PCS 20 (128%), *L. plantarum* PCS 26 (107%) and *L. rhamnosus* GG (113%), while a reduction was seen with heat-killed *L. acidophilus* (77%). The effect of all the cell-free probiotic supernatants led to elevated SGLT1 protein levels in Caco-2 cells, confirming the *SGLT1* gene transcript results (Figure 2b and Figure S7). Specifically, *L. plantarum* PCS 26, *L. rhamnosus* GG and *L. acidophilus* cell-free supernatants significantly increased SGLT1 protein expression to 190% (*p* < 0.001), 210% (*p* < 0.001) and 230% (*p* < 0.001), respectively. An increase in SGLT1 protein level (122%) was also observed after *L. plantarum* PCS 20 cell-free supernatant exposure, although this effect was not statistically significant.

- GLUT2

**Figure 5:**
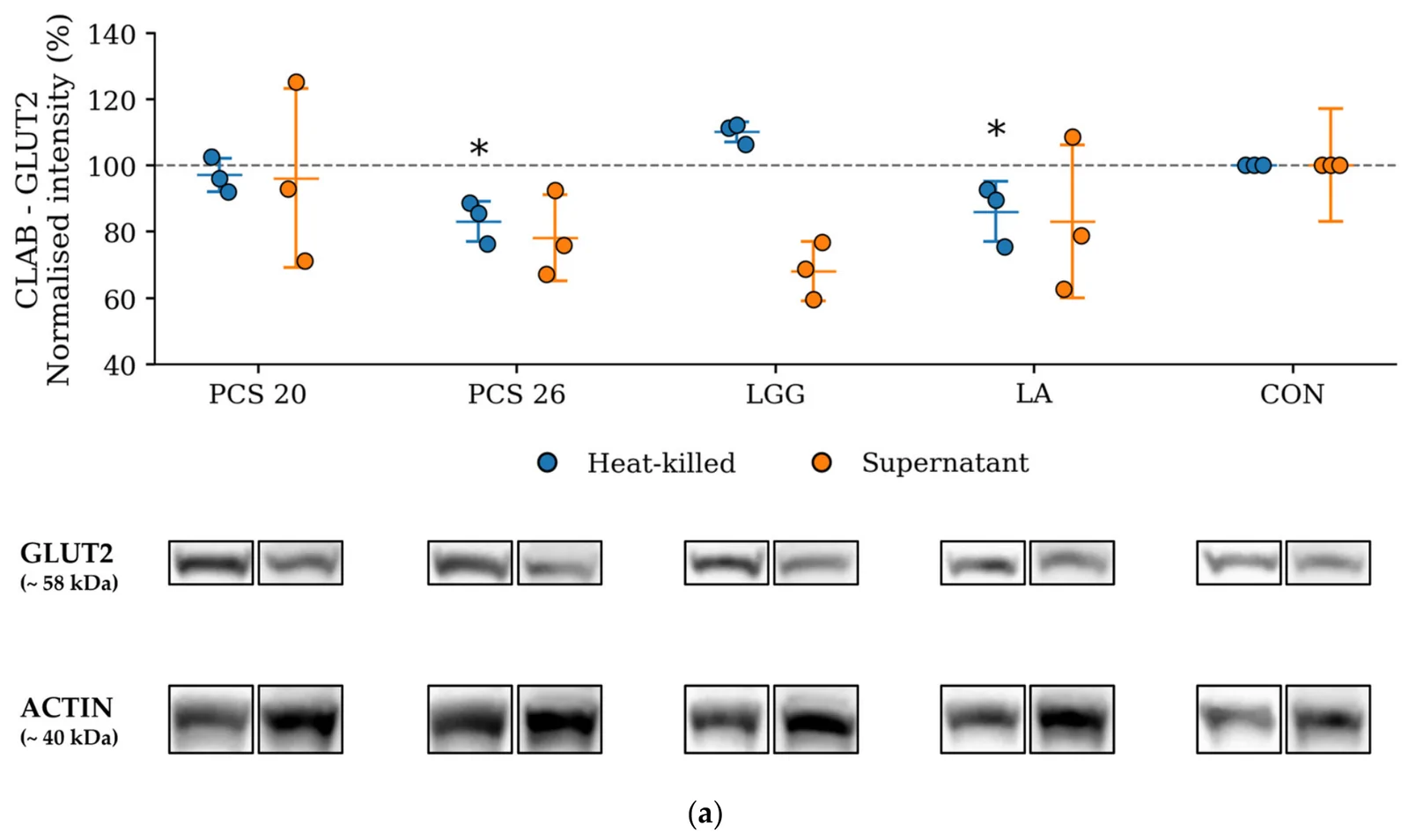

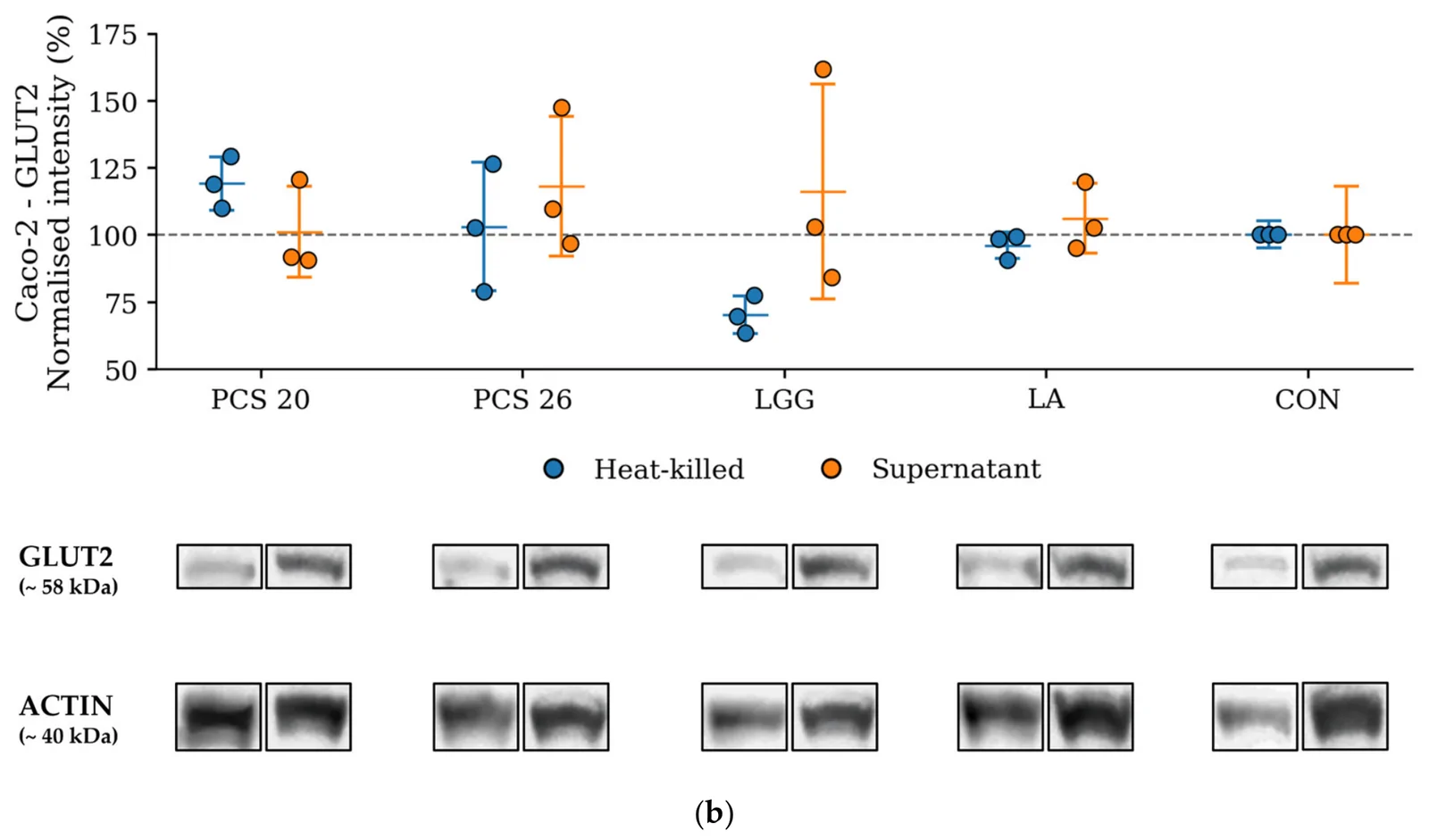
GLUT2 protein expression in CLAB (**a**) and Caco-2 (**b**) cells after 6 h of incubation with heat-killed probiotic strains and cell-free supernatants of the probiotic strains *Lactiplantibacillus plantarum* PCS 20 (PCS 20), *Lactiplantibacillus plantarum* PCS 26 (PCS 26), *Lacticaseibacillus rhamnosus* GG (LGG), and *Lactobacillus acidophilus* (LA). GLUT2 band intensities were normalised to the reference protein ACTIN and expressed as percentages relative to the corresponding control (CON), which was set to 100%. Data are presented as mean ± SD from three independent biological experiments (*n* = 3). Statistical differences compared with the corresponding control were determined using Welch’s ANOVA followed by Dunnett’s T3 post hoc test (* *p* < 0.05). Representative immunoblot bands are shown for each experimental condition. Vertical lines indicate the boundaries between non-adjacent lanes originating from two separate immunoblots corresponding to the heat-killed probiotic and cell-free supernatant experiments. GLUT2 and ACTIN were detected on the same membrane. GLUT2: facilitative glucose transporter 2.

Figure 5a shows the results of GLUT2 protein expression in CLAB cells after exposure to heat-killed probiotic strains and cell-free probiotic supernatants (Figures S8 and S9, respectively). After exposure to heat-killed *L. plantarum* PCS 20, *L. plantarum* PCS 26 and *L. acidophilus* probiotic strains, GLUT2 protein levels decreased, consistent with the gene expression results (Figure 3a). In fact, the most potent and statistically significant reduction in GLUT2 was observed after heat-killed *L. plantarum* PCS 26 addition (83%; *p* = 0.016), followed by *L. acidophilus* (86%; *p* = 0.037) and *L. plantarum* PCS 20 (97%). The reduction with *L. plantarum* PCS 20 was not statistically significant. However, the GLUT2 protein level was increased after exposure to *L. rhamnosus* GG (111%), although this did not confirm the GLUT2 gene transcript result (Figure 3a). The cell-free supernatants of all probiotic strains (Figure 5a and Figure S9) reduced GLUT2 protein levels in CLAB cells, with the *L. rhamnosus* GG cell-free supernatant showing the most potent effect (68%), followed by *L. plantarum* PCS 26 (78%), *L. acidophilus* (83%) and *L. plantarum* PCS 20 (96%). These results were not statistically significant, but consistent with the *GLUT2* gene expression results (Figure 3a).

In Caco-2 cells (Figure 5b and Figure S10), heat-killed *L. plantarum* PCS 26 and *L. plantarum* PCS 20 increased GLUT2 levels (103% and 119%, respectively); however, the latter did not confirm the gene results, where minimal downregulation of *GLUT2* gene was observed (Figure 3b). Furthermore, heat-killed *L. acidophilus* and *L. rhamnosus* GG reduced GLUT2 protein levels to 96% and 70%, respectively. However, a minimal upregulation of the *GLUT2* gene was observed after *L. rhamnosus* GG addition (Figure 3b). An increase in *GLUT2* gene expression after exposure to *L. plantarum* PCS 26, *L. rhamnosus* GG and *L. acidophilus* cell-free supernatants (Figure 3b) resulted in increased GLUT2 protein levels (118%, 116%, and 106%, respectively) (Figure 5b and Figure S11). In contrast, the *L. plantarum* PCS 20 cell-free supernatant appeared to have a minor effect on *GLUT2* gene downregulation (Figure 3b), which was not consistent with a minor increase in GLUT2 protein level (101%) (Figure 5b and Figure S11). None of the observed changes were statistically significant.

### 3.5. Analysis of Glucose Concentration in the Basolateral Compartment

**Figure 6:**
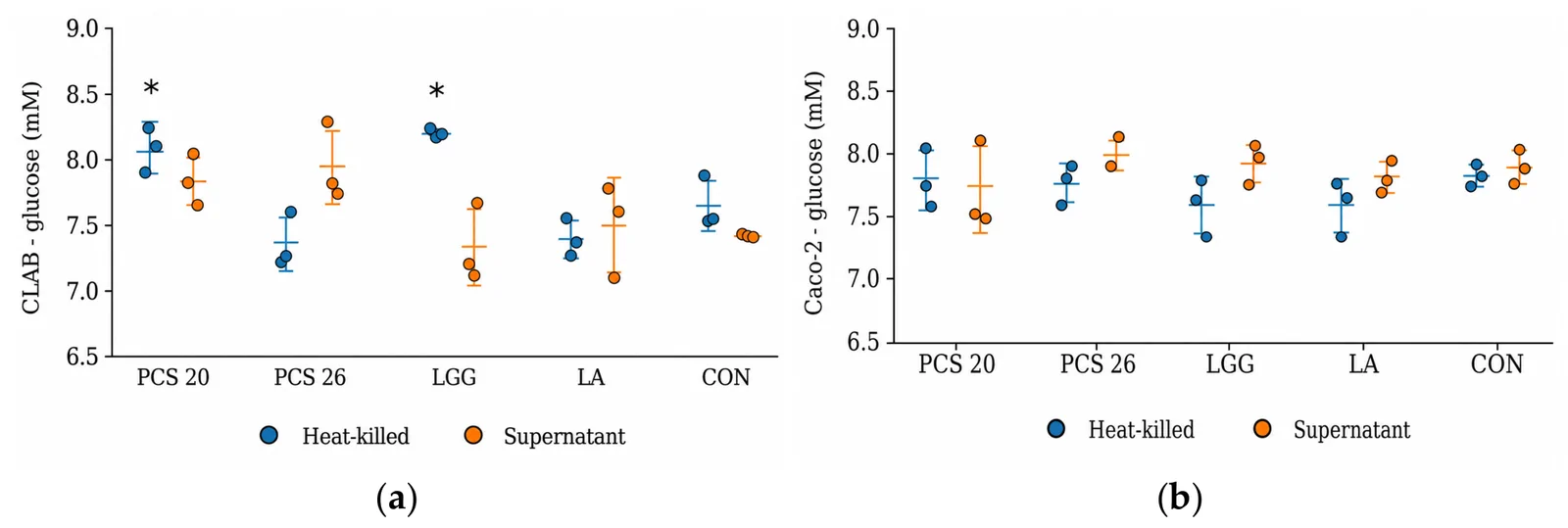
Glucose concentration in the basolateral compartment of polarized CLAB and Caco-2 cell monolayers (**a**,**b**) respectively, after co-culture with the probiotic strains *Lactiplantibacillus plantarum* PCS 20 (PCS 20), *Lactiplantibacillus plantarum* PCS 26 (PCS 26), *Lacticaseibacillus rhamnosus* GG (LGG), and *Lactobacillus acidophilus* (LA). Glucose was added to the apical compartment, and its concentration was determined in the basolateral compartment following a 6-h incubation. Data are presented as mean ± SD from three independent biological experiments (*n* = 3). Statistical differences in comparison with the corresponding control (CON) were determined using one-way ANOVA followed by Dunnett’s post hoc test (* *p* < 0.05).

To account for potential glucose uptake or metabolism by the probiotic bacteria, glucose concentrations were measured in parallel after incubation with bacteria alone (Figure S12). None of the tested probiotic strains showed a statistically significant difference compared with the probiotic-free control, suggesting that direct bacterial glucose utilisation did not substantially contribute to the observed changes in glucose concentration. Glucose concentration was measured in the basolateral compartment of polarised CLAB and Caco-2 epithelial monolayers cultured on microporous membranes. Glucose was administered to the apical side of the membrane, and its concentration in the basolateral compartment was quantified following a 6-h incubation period (Figure 6). In both cell lines, similar trends were observed for glucose concentration and GLUT2 protein expression (Figure 5).

In CLAB cells (Figure 6a), glucose did not decrease significantly compared with the control (7.62 mM) after treatment with heat-killed *L. plantarum* PCS 26 (7.34 mM) and *L. acidophilus* (7.40 mM). However, a statistically significant increase in glucose concentration was observed after treatment with heat-killed *L. plantarum* PCS 20 (8.02 mM; * *p* = 0.030) and *L. rhamnosus* GG (8.12 mM; * *p* = 0.006). Compared to the control (7.55 mM), two cell-free supernatants of live probiotics slightly increased glucose concentration (*L. plantarum* PCS 20—7.82 mM; *L. plantarum* PCS 26—7.91 mM), whereas *L. rhamnosus* GG (7.32 mM) and *L. acidophilus* (7.48 mM) decreased glucose concentration. Nevertheless, none of these effects were statistically significant.

Changes in glucose concentration compared with the control in Caco-2 cells (Figure 6b) were not statistically significant for either heat-killed or supernatant samples. However, heat-killed probiotics generally decreased glucose concentration, with the lowest glucose concentration observed after the addition of heat-killed *L. acidophilus* (7.60 mM) compared to the control (7.81 mM). In contrast, the cell-free supernatant of *L. plantarum* PCS 20 decreased the glucose concentration the most (7.74 mM), followed by *L. acidophilus* (7.78 mM), *L. rhamnosus* GG (7.91 mM) and *L. plantarum* PCS 26 (7.95 mM), which showed a slight increase in glucose concentration compared to the control.

To provide an area-normalised estimate of glucose accumulation in the basolateral compartment, an exploratory calculation of 6-h net basolateral glucose accumulation, normalised to membrane area (µmol/cm^2^), was performed (Table S1). In CLAB cells, heat-killed PCS 20 and LGG significantly increased 6-h net basolateral glucose accumulation compared with the control, whereas no individual probiotic treatment differed significantly from the control in Caco-2 cells.

Furthermore, associations between glucose concentration and SGLT1 or GLUT2 protein expression were assessed. Across Caco-2 cells, no significant associations were observed between basolateral glucose concentration in either SGLT1 or GLUT2 protein expression following heat-killed bacterial or cell-free supernatant treatments (all *p* > 0.05). In CLAB cells, basolateral glucose concentration was positively associated with GLUT2 protein expression following heat-killed bacterial treatment (Pearson r = 0.731, 95% CI 0.349–0.904, *p* = 0.002; *n* = 15) and with SGLT1 protein expression following cell-free supernatant treatment (Spearman ρ = 0.560, 95% CI 0.120–0.837, *p* = 0.030; *n* = 15). The remaining associations were not statistically significant (Tables S2–S4; Figures S13–S15).

## 4. Discussion

The maintenance of glucose homeostasis is essential for the physiological function of every cell [84]. With the growing recognition of the microbiota’s role in the pathogenesis of most metabolic diseases, the importance of probiotic bacteria in glucose homeostasis and glycaemic control has increased. Numerous studies have highlighted the effects of various probiotic bacteria on glucose, related metabolism, and overall homeostasis [14,15,18,32,85], but few have focused on mechanisms of action at the molecular level. When molecular mechanisms have been explored, they are usually limited to hormonal-immune signalling pathways in the gut or other tissues. Some studies have examined the effects of probiotics on tissue-specific glucose transporters at the molecular level [17]. Although many studies have described the beneficial effects of different probiotic species, in recent years paraprobiotics and postbiotics have gained attention because of several potential advantages over live probiotics. Therapeutically, they provide a safer option for individuals with weakened immune systems, premature infants, and low birth weight babies, without lasting effects if therapy is discontinued [30,31,32,36]. In industrial biotechnology and food safety, they do not interact with food components, can be heat-killed during thermal processing or added afterwards without risk of contamination, and their non-viable nature may result in longer shelf life and greater stability. Additionally, their transport and storage are more convenient, and they allow for better biofilm preservation and control [29,86].

Porcine and human cell lines were selected mainly because pigs are considered a highly relevant experimental model for mechanistic and functional studies, and because of their close similarity to humans in terms of intestinal morphology, histology, and nutrient transport physiology [87,88]. In contrast, Caco-2 cells are derived from a human colorectal adenocarcinoma; nevertheless, following differentiation at late confluency, they exhibit many morphological and functional characteristics of mature intestinal enterocytes [89,90,91,92]. Owing to these features, Caco-2 cells have been extensively employed as an in vitro model to evaluate the biological effects of Lactobacillaceae-derived probiotics [93,94,95,96]. The differential responses observed between CLAB and Caco-2 cells may reflect differences in host–cell phenotype rather than species differences alone. Although both models exhibit characteristics of intestinal epithelial cells, they differ substantially in cellular origin and differentiation status. CLAB represents a non-carcinogenic porcine-derived intestinal epithelial model [62,63,64,65], whereas Caco-2 cells originate from a human colorectal adenocarcinoma and acquire an enterocyte-like phenotype during post-confluent differentiation [89,90,91]. Importantly, differentiation of Caco-2 cells is accompanied by changes in the expression and functional activity of intestinal transporters, including SGLT1 and GLUT2, and the expression profile of differentiated Caco-2 cells can still differ considerably from that of native human intestinal tissue [97]. Furthermore, it has been shown that species-related differences exist in the distribution and regulation of intestinal glucose transporters [12]. Another study examining the porcine small intestine found that SGLT1 and GLUT2 expression varies along the intestinal axis and that functional glucose transport differs between jejunum and ileum even when transporter abundance is similar, which suggests that post-translational regulation, phosphorylation and membrane localization may also contribute to functional regulation [98]. Moreover, direct comparisons of porcine and human intestinal epithelial cell models have demonstrated that the same microbial stimulus can induce different epithelial responses, suggesting that species, cellular origin and cell-specific signalling characteristics may all contribute to the observed differences [99]. Thus, the cell-line-dependent effects observed in the present study, particularly the more pronounced modulation of GLUT2 in CLAB cells, may reflect differences in the basal differentiation state and regulatory machinery governing intestinal glucose transport rather than a uniform response to the tested Lactobacillaceae-derived components.

SGLT1 is a 70–73 kDa membrane protein encoded by the *SLC5A1* gene, with high affinity for glucose and galactose binding but low transport capacity [100]. Transport occurs via secondary Na+/K+ ATPase-dependent active transport [2,7]. In humans, SGLT1 was first identified in the small intestine, where it also predominates at the whole organism level [101]. The important role of SGLT1 in glucose absorption in the small intestine has also been demonstrated in pigs [10]. In fact, SGLT1 gene expression is believed to be most intense at birth and during suckling [9].

In our study, the addition of all heat-killed probiotics and all cell-free probiotic supernatants up-regulated *SGLT1* in CLAB cells. The trend could also be observed at the protein level, except for the heat-killed *L. acidophilus*, where a slight decrease in protein level was detected. It is already known that protein expression can sometimes differ from gene expression. This phenomenon arises because there are many regulatory layers between transcription and functional protein and these can buffer, amplify, or even invert the effects of changes in mRNA levels. The reduction in protein level after upregulation in gene expression may be due to translational repression, increased protein degradation, translational buffering/compensation, alternative transcripts or non-productive isoforms [102,103,104]. It also appears that the cell-free probiotic supernatants of all strains have a greater effect on the SGLT1 protein level than the heat-killed probiotics. Interestingly, in our recent study [22], we demonstrated a statistically significant up-regulation of *SGLT1* by *L. rhamnosus* GG addition in CLAB cells. After observing the results of this study, the findings suggest that the effect of *L. rhamnosus* GG may be more attributable to the supernatant particles of the viable strain rather than the non-viable strain. *Lacticaseibacillus rhamnosus* is a well-studied species, and different strains have demonstrated positive glycaemic effects on various blood parameters in in vivo mouse and rat animal studies. Among these, reductions in postprandial glucose [105], fasting plasma glucose [23,105,106], improvement in HOMA-IR [105], increases in insulin levels and insulin sensitivity [106], and improvements in glucose tolerance [23,106,107,108] have been reported. In a study on obese mice, an improvement and reduction in glucose hypersensitivity and insulin resistance in adipose tissue after the addition of *Lactobacillus rhamnosus* GG was observed [108]. A study on intestinal porcine epithelial cells [109] showed improved barrier function, increased intracellular reactive oxygen species production, a proinflammatory cytokine response, and inhibition of adhesion after *Lactobacillus rhamnosus* addition. Moreover, two studies on piglet models [110,111] have shown that supplementation with *Lacticaseibacillus rhamnosus* had a positive effect on small intestinal tissue maturation and immunity, and caused significant changes in the caecal microbiome, respectively.

In Caco-2 cells, the cell-free supernatants of all probiotic strains up-regulated *SGLT1*, as well as its protein expression. Moreover, the addition of *L. acidophilus*, *L. rhamnosus* GG and *L. plantarum* PCS 26 cell-free supernatants significantly increased SGLT1 protein levels. There was also a minor increase in SGLT1 protein levels after treatment with heat-killed probiotic samples (*L. plantarum* PCS 20, *L. plantarum* PCS 26, and *L. rhamnosus* GG), except for *L. acidophilus*, where the biggest reduction in protein levels was observed, although this was not statistically significant. Notably, in our previous study [22], we observed a minor up-regulation of the *SGLT1* gene following the addition of all viable probiotics. Rooj et al. [19] and Uhlig et al. [54] have suggested that the addition of certain *Lactobacillus* strain supernatants can affect *SGLT1* expression and glucose uptake. However, it is already known that in in vitro studies, the origin of Caco-2 cells plays a heterogeneous role in glucose uptake by the SGLT1 transporter, which also differs from in vivo studies, and should be taken into account [112,113,114].

GLUT2 is a 57 kDa molecule encoded by the *SLC2A2* gene. The cDNA sequence was first cloned from rat and later from human liver [115,116]. Among all GLUTs, GLUT2 uniquely has a low glucose binding affinity [2,117]. *GLUT2* expression in cells is usually very high, so the rate of glucose absorption does not limit glucose utilisation. In the intestine, GLUT2 is located on the basolateral membrane of epithelial cells (enterocytes), where it is responsible for glucose transport from the cell [118]. However, its function may be dispensable during normal intestinal glucose absorption [119,120,121,122,123,124,125,126].

The GLUT2 protein level in CLAB cells was decreased by all the heat-killed probiotics and the cell-free probiotic supernatants, confirming the gene expression results. The only exception was the effect of heat-killed *L. rhamnosus* GG, which, after causing minor *GLUT2* downregulation, did not significantly increase the GLUT2 protein level. This phenomenon is less common, but can be explained by several mechanisms resulting in protein-centric regulation, such as a longer protein half-life than that of its mRNA, increased translational efficiency, and reduced protein degradation due to suppressed proteasomal or lysosomal activity [127,128,129]. In CLAB cells, glucose concentration in the basolateral compartment also appeared to change after supplementation with heat-killed probiotics. Additionally, a strong, statistically significant correlation between GLUT2 protein expression and glucose concentration was observed. Consistent with these findings, the area-normalised analysis confirmed a significant increase in 6-h net basolateral glucose accumulation following treatment with heat-killed *L. plantarum* PCS 20 and *L. rhamnosus* GG compared with the control. However, these functional changes did not uniformly parallel changes in GLUT2 protein expression across individual strains, suggesting that basolateral glucose accumulation cannot be attributed solely to changes in GLUT2 abundance. Interestingly, supplementation with heat-killed *L. plantarum* PCS 20 seemed to influence *GLUT2* downregulation, but appeared to significantly increase glucose concentration. This observation may indicate the involvement of an alternative mechanism in transepithelial glucose transport. Studies conducted in *GLUT2*-deficient mice have suggested the existence of an additional transepithelial glucose transport pathway that may be mediated by membrane trafficking involving the endoplasmic reticulum in hepatocytes and enterocytes [120,130,131]. When compared, the cell-free supernatants of all probiotic strains were more effective than the heat-killed probiotics at decreasing GLUT2 protein levels, and partly resulted in a decreased glucose concentration as well. In fact, compared to the control, the cell-free supernatants from *L. rhamnosus* GG and PCS 26 also downregulated *GLUT2* the most; however, these effects were not statistically significant. In our previous study [22], we demonstrated a statistically significant downregulation of *GLUT2* by viable *L. plantarum* PCS 26, and the results of this study suggest that this effect could be partly due to the supernatants of the viable *L. plantarum* PCS 26 as well. *L. plantarum* PCS 26 has already attracted attention as a regulator of cholesterol homeostasis in a pig cell model [68], promoted gut health in a small intestinal cell model [67], showed supportive effects by alleviating metabolic syndrome symptoms and reducing cardiovascular disease risks in a pilot clinical study [73], decreased glucose transport by downregulating *GLUT2* expression in pig epithelial cells [22], and showed promising results in alleviating urinary tract infection symptoms and in their prevention in a pilot study in children [132]. Nevertheless, none of the mentioned studies used cell-free probiotic supernatants or heat-killed strains in their experiments.

In contrast, in Caco-2 cells, cell-free supernatants of probiotic strains surprisingly increased *GLUT2* gene transcripts and GLUT2 protein levels, except for *L. plantarum* PCS 20. In addition, a similar trend appeared in the glucose concentration results for *L. plantarum* PCS 26 and *L. rhamnosus* GG, although it was not statistically significant. Similarly, heat-killed *L. plantarum* PCS 20 and *L. plantarum* PCS 26 increased GLUT2 protein levels. The increase observed with *L. plantarum* PCS 20 was not consistent with the corresponding gene expression result, whereas *L. rhamnosus* GG and *L. acidophilus* decreased GLUT2 protein levels. In our previous study [22], we showed that the addition of all viable Lactobacillaceae strains tended to downregulate the *GLUT2* expression, with *L. acidophilus* showing the greatest effect. However, in this study, heat-killed *L. acidophilus* and *L. rhamnosus* GG decreased GLUT2 protein levels, which was also reflected in a slight, although not significant, decrease in glucose concentration. Moreover, the effect of heat-killed *L. acidophilus* also decreased the SGLT1 protein levels, demonstrating the potential dual effect of *L. acidophilus* on both glucose transporters. Interestingly, in our previous study [22] we showed that the addition of viable *L. acidophilus* also most significantly decreased GLUT2 protein levels. In fact, many human and animal studies have already shown the beneficial, hypoglycaemic and anti-diabetic role of *L. acidophilus* alone or in combination with other probiotic strains [17], but the role of postbiotics [35,37,55,133] and paraprobiotics [60,77,133] in glucose metabolism and homeostasis in humans and human cell models is still in its infancy and requires further research.

## 5. Conclusions

The results of this study show that the effects of the tested preparations (heat-killed probiotics and cell-free supernatants of viable strains) are both strain-specific and cell-line-specific. We observed that these preparations generally increase SGLT1 protein levels rather than downregulate them in both epithelial cell lines, except for heat-killed *L. acidophilus*. However, these effects varied among treatments and between cell models. A potential effect of the selected cell-free probiotic supernatants on *GLUT2* downregulation, GLUT2 protein expression, and decreased glucose concentration was observed primarily in CLAB cells. To a lesser extent, this effect was also observed in Caco-2 cells, with heat-killed *L. acidophilus* and *L. rhamnosus* GG showing the most promising results. It should be noted, however, that the preparation of heat-killed probiotics and cell-free supernatants of viable probiotics still varies among different studies [30,31,36], which may significantly influence the results. In addition, to exclude changes in other metabolites or medium properties, a comprehensive chemical characterisation of the medium and supernatants is needed.

Despite the limitations inherent to in vitro models, further studies using differentiated human intestinal organoids or enteroids are needed to confirm these results. The observed changes in SGLT1 and GLUT2 protein expression and basolateral glucose appearance may suggest a potential role of these transporters in the modulation of intestinal glucose transport by probiotic-derived preparations. However, the underlying mechanisms remain to be established, as intracellular glucose accumulation, transporter localization, and pathway-specific transport were not assessed in the present study. Although changes in glucose transporter expression could be a likely cause of glucose accumulation in the basolateral region, these changes alone cannot establish that SGLT1 or GLUT2 mediates the measured glucose movement. To draw a more precise mechanistic conclusion, transporter-selective inhibitor assays or *SLC5A1/SLC2A2* knockdown experiments would be required. Furthermore, GLUT2 activity and its contribution to intestinal glucose transport are closely related to its intracellular trafficking and membrane localization, but GLUT2 subcellular localization and membrane trafficking were beyond the scope of the present study. Further studies employing approaches such as surface biotinylation, membrane fractionation, or confocal immunofluorescence will be required to determine whether the observed changes in GLUT2 expression are accompanied by alterations in its membrane localization and trafficking. Additionally, the bioaccessibility and bioavailability of promising biomolecules in in vivo studies need to be thoroughly and more effectively addressed.

## Figures and Tables

**Table 1:** Primer sequences, species, NCBI RefSeq accession numbers, and amplicon sizes used for qPCR analysis.

Gene	Species	Forward Primer (5′–3′)	Reverse Primer (5′–3′)	Amplicon Size (bp)	NCBI RefSeq Accession Number(s)
*B2M*	*Sus scrofa*	CAAGATAGTTAAGTGGGATCG	TGGTAACATCAATACGATTTC	161	Reference [82]; accession number not reported
*POLR2A*	*Sus scrofa*	ACCATCAAGCGAGTGCAGTT	TCGGTTGTCTCTGGGTATTTG	95	XM_005669224.1
SGLT1	*Sus scrofa*	AGTGGGCAGCTCTTCGATTA	CCAGCCCAATCATACATCCT	148	NM_001164021.1
GLUT2	*Sus scrofa*	ATTCTTTGGTGGGATGCTTG	ATGAGATGGTCCCAATTTCG	118	NM_001097417.1
*ACTB*	*Homo* *sapiens*	CTCTTCCAGCCTTCCTTCCT	AGCACTGTGTTGGCGTACAG	116	NM_001101.3
*POLR2A*	*Homo* *sapiens*	CTTCACGGTGCTGGGCATT	GTGCGGCTGCTTCCATAA	239	NM_000937.4
SGLT1	*Homo* *sapiens*	ATGATCAGCCGCATTCTGTA	AGGTTGGATAGGCGATGTTG	109	NM_000343.3; NM_001256314.1
GLUT2	*Homo* *sapiens*	CATGTCAGTGGGACTTGTGC	AAACTCAGCCACCATGAACC	133	NM_000340.1; NM_001278658.1; NM_001278659.1

*B2M*: beta-2-microglobulin; *ACTB*: actin beta; *POLR2A*: RNA polymerase II subunit A; SGLT1: sodium/glucose cotransporter 1 (SLC5A1); GLUT2: glucose transporter 2 (*SLC2A2*). For porcine *B2M*, the primer pair was adopted from reference [82].

## Data Availability

Dataset available on request from the authors.

## Supplementary Materials

The following supporting information can be downloaded at: https://www.mdpi.com/article/10.3390/nu18172830/s1, Figure S1. Preliminary immunoblot showing SGLT1 and ACTIN detection in CLAB and Caco-2 cell lysates. Figure S2. Preliminary immunoblot showing GLUT2 and ACTIN detection in CLAB and Caco-2 cell lysates. Figure S3: Expression of reference genes in CLAB (a) and Caco-2 (b) cells. Figure S4. Immunoblot showing SGLT1 and ACTIN protein expression in CLAB cells after 6 h of incubation with heat-killed probiotic strains *Lactiplantibacillus plantarum* PCS 20 (PCS 20), *Lactiplantibacillus plantarum* PCS 26 (PCS 26), *Lacticaseibacillus rhamnosus* GG (LGG), and *Lactobacillus acidophilus* (LA), and the corresponding control (CON). Figure S5. Immunoblot showing SGLT1 and ACTIN protein expression in CLAB cells after 6 h of incubation with cell-free supernatants of the probiotic strains *Lactiplantibacillus plantarum* PCS 20 (PCS 20), *Lactiplantibacillus plantarum* PCS 26 (PCS 26), *Lacticaseibacillus rhamnosus* GG (LGG), and *Lactobacillus acidophilus* (LA), and the corresponding control (CON). Figure S6. Immunoblot showing SGLT1 and ACTIN protein expression in Caco-2 cells after 6 h of incubation with heat-killed probiotic strains *Lactiplantibacillus plantarum* PCS 20 (PCS 20), *Lactiplantibacillus plantarum* PCS 26 (PCS 26), *Lacticaseibacillus rhamnosus* GG (LGG), and *Lactobacillus acidophilus* (LA), and the corresponding control (CON). Immunoblot showing SGLT1 and ACTIN protein expression in Caco-2 cells after 6 h of incubation with cell-free supernatants of the probiotic strains *Lactiplantibacillus plantarum* PCS 20 (PCS 20), *Lactiplantibacillus plantarum* PCS 26 (PCS 26), *Lacticaseibacillus rhamnosus* GG (LGG), and *Lactobacillus acidophilus* (LA), and the corresponding control (CON). Figure S7. Immunoblot showing SGLT1 and ACTIN protein expression in Caco-2 cells after 6 h of incubation with cell-free supernatants of the probiotic strains *Lactiplantibacillus plantarum* PCS 20 (PCS 20), *Lactiplantibacillus plantarum* PCS 26 (PCS 26), *Lacticaseibacillus rhamnosus* GG (LGG), and *Lactobacillus acidophilus* (LA), and the corresponding control (CON). SGLT1 and ACTIN were detected at approximately 72 kDa and 42 kDa, respectively. Three independent biological experiments (*n* = 3) are shown for each experimental condition. Bands not included in the final manuscript figure are marked with an “X”. Rectangles indicate the SGLT1 and corresponding ACTIN bands selected as representative bands for presentation in Figure 4b of the manuscript. SGLT1: sodium-driven glucose transporter 1. Figure S8. Immunoblot showing GLUT2 and ACTIN protein expression in CLAB cells after 6 h of incubation with heat-killed probiotic strains *Lactiplantibacillus plantarum* PCS 20 (PCS 20), *Lactiplantibacillus plantarum* PCS 26 (PCS 26), *Lacticaseibacillus rhamnosus* GG (LGG), and *Lactobacillus acidophilus* (LA), and the corresponding control (CON). Figure S9. Immunoblot showing GLUT2 and ACTIN protein expression in CLAB cells after 6 h of incubation with cell-free supernatants of the probiotic strains *Lactiplantibacillus plantarum* PCS 20 (PCS 20), *Lactiplantibacillus plantarum* PCS 26 (PCS 26), *Lacticaseibacillus rhamnosus* GG (LGG), and *Lactobacillus acidophilus* (LA), and the corresponding control (CON). Figure S10. Immunoblot showing GLUT2 and ACTIN protein expression in Caco-2 cells after 6 h of incubation with heat-killed probiotic strains *Lactiplantibacillus plantarum* PCS 20 (PCS 20), *Lactiplantibacillus plantarum* PCS 26 (PCS 26), *Lacticaseibacillus rhamnosus* GG (LGG), and *Lactobacillus acidophilus* (LA), and the corresponding control (CON). Figure S11. Immunoblot showing GLUT2 and ACTIN protein expression in Caco-2 cells after 6 h of incubation with cell-free supernatants of the probiotic strains *Lactiplantibacillus plantarum* PCS 20 (PCS 20), *Lactiplantibacillus plantarum* PCS 26 (PCS 26), *Lacticaseibacillus rhamnosus* GG (LGG), and *Lactobacillus acidophilus* (LA), and the corresponding control (CON). Figure S12. Glucose concentration measured after 6 h of incubation of the probiotic strains *Lactiplantibacillus plantarum* PCS 20 (PCS 20), *Lactiplantibacillus plantarum* PCS 26 (PCS 26), *Lacticaseibacillus rhamnosus* GG (LGG), and *Lactobacillus acidophilus* (LA) in DMEM cell culture medium. Figure S13. Correlation between basolateral glucose concentration and SGLT1 or GLUT2 protein expression in Caco-2 and CLAB cells following treatment with heat-killed probiotics or their cell-free supernatants. Figure S14. Treatment-level distribution of basolateral glucose concentration and SGLT1 and GLUT2 protein expression in CLAB cells following treatment with heat-killed probiotics or their cell-free supernatants. Figure S15. Treatment-level distribution of basolateral glucose concentration and SGLT1 and GLUT2 protein expression in Caco-2 cells following treatment with heat-killed probiotics or their cell-free supernatants. Table S1. 6-h net basolateral glucose accumulation by probiotic treatment. Table S2. Individual paired biological observations used for correlation analyses between basolateral glucose concentration and SGLT1 or GLUT2 protein expression. Table S3. Correlation analyses between basolateral glucose concentration and SGLT1 or GLUT2 protein expression. Table S4. Treatment-level distribution of basolateral glucose concentration and SGLT1 and GLUT2 protein expression.). Raw data (15 excel files).

## Abbreviations

The following abbreviations are used in this manuscript:

SGLT1	sodium-driven glucose cotransporter 1
GLUT2	facilitative glucose transporter 2
CLAB	non-carcinogenic porcine-derived enterocytes
STR	short tandem repeat
PCR	polymerase chain reaction
PAS	periodic acid-Schiff
DMEM	Dulbecco’s modified Eagle’s medium
FBS	foetal bovine serum
MRS	De man Rogosa, Sharpe
MTT	3-(4,5-dimethylthiazol-2-yl)-2, 5-diphenyltetrazolium bromide
cDNA	complementary deoxyribonucleic acid
mRNA	messenger ribonucleic acid
qPCR	quantitative real-time polymerase chain reaction
B2M	beta-2-microglobulin
POLR2A	polymerase II subunit A
ACTB	actin beta
SDS-PAGE	sodium dodecyl sulfate-polyacrylamide gel electrophoresis
