# Supplementary material for: Differential Regulation of Intestinal Glucose Transporters SGLT1 and GLUT2 by Lactobacillaceae-Derived Heat-Killed Probiotics and Cell-Free Probiotic Supernatants
Source: Nutrients. 2026 Aug 28;18(17):2830. doi: 10.3390/nu18172830 (PMC13567212; doi:10.3390/nu18172830)
Supplement: Supplementary file 1 [file nutrients-18-02830-s001.zip › nutrients-4485338-supplement.pdf]

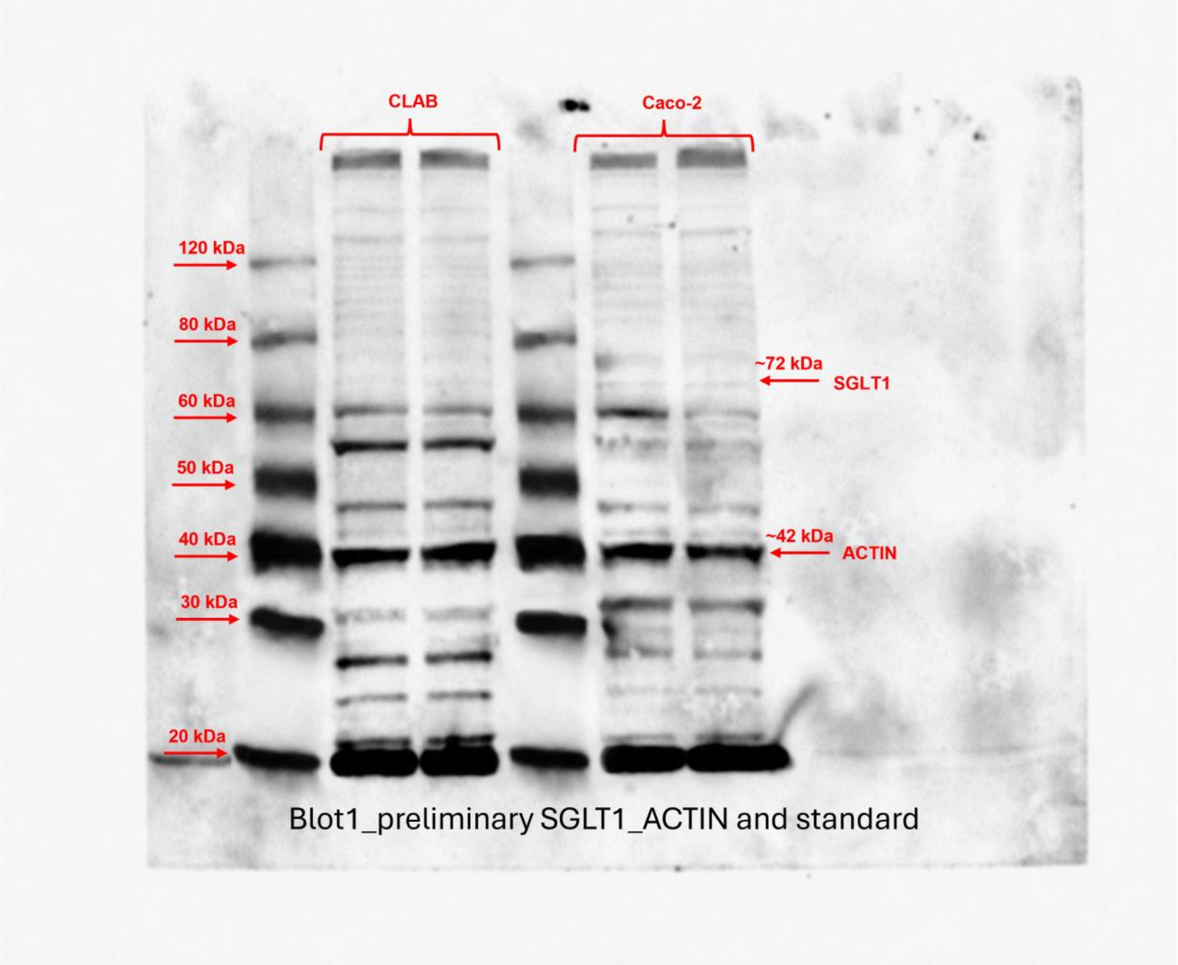

Figure S1. Preliminary immunoblot showing SGLT1 and ACTIN detection in CLAB and Caco-2 cell lysates. A molecular-weight protein standard was included to assess the apparent molecular weights of the detected immunoreactive bands, with marker positions indicated at 20, 30, 40, 50, 60, 80, and 120 kDa. SGLT1 and ACTIN were detected at approximately 72 kDa and 42 kDa, respectively. The membrane shown was stripped and reprobed for detection of the target and reference proteins. CLAB: non-carcinogenic porcine-derived enterocytes; Caco-2: human epithelial colorectal adenocarcinoma cells; SGLT1: sodium-driven glucose transporter 1.

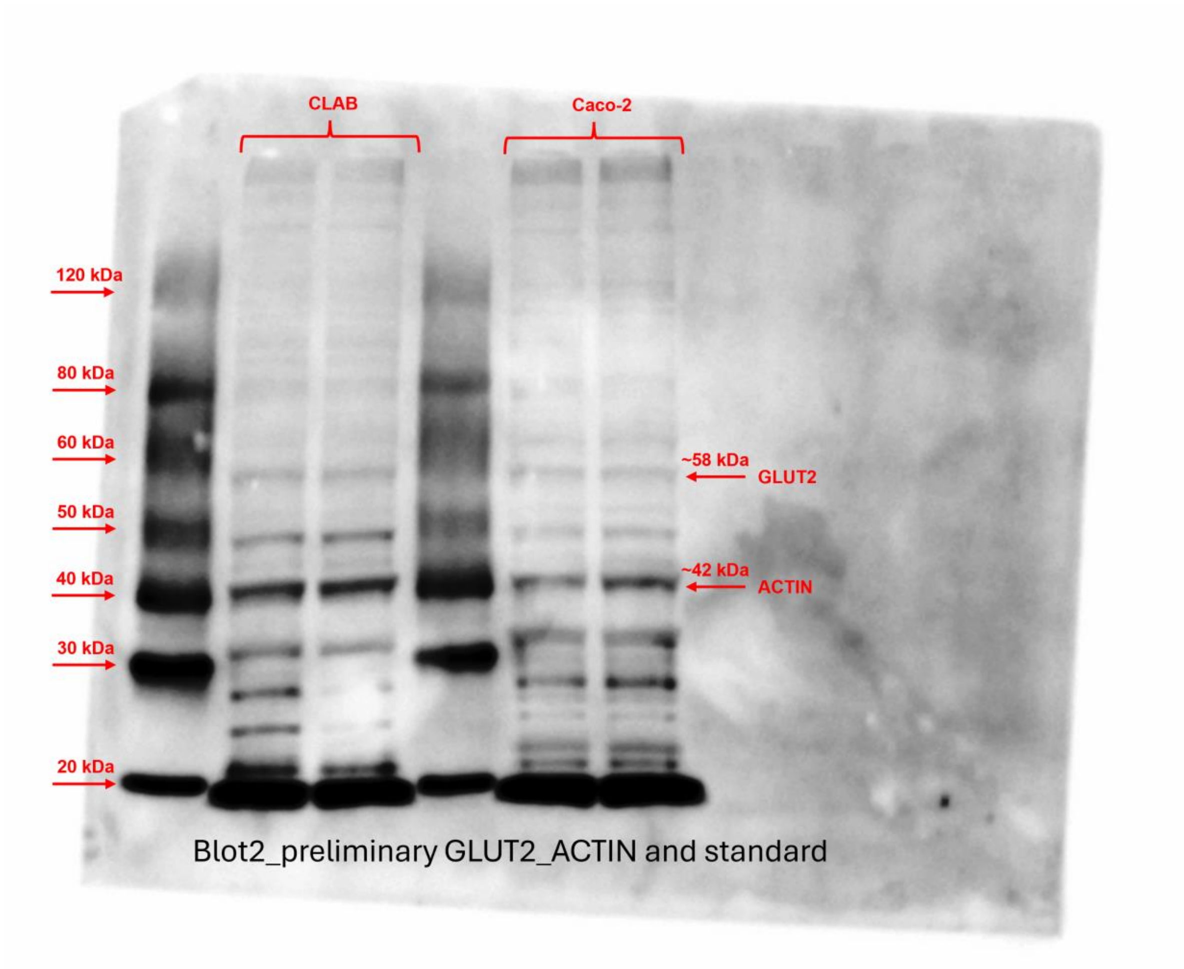

Figure S2. Preliminary immunoblot showing GLUT2 and ACTIN detection in CLAB and Caco-2 cell lysates. A molecular-weight protein standard was included to assess the apparent molecular weights of the detected immunoreactive bands, with marker positions indicated at 20, 30, 40, 50, 60, 80, and 120 kDa. GLUT2 and ACTIN were detected at approximately 58 kDa and 42 kDa, respectively. The membrane shown was stripped and reprobed for detection of the target and reference proteins. CLAB: non-carcinogenic porcine-derived enterocytes; Caco-2: human epithelial colorectal adenocarcinoma cells; GLUT2: facilitative glucose transporter 2.

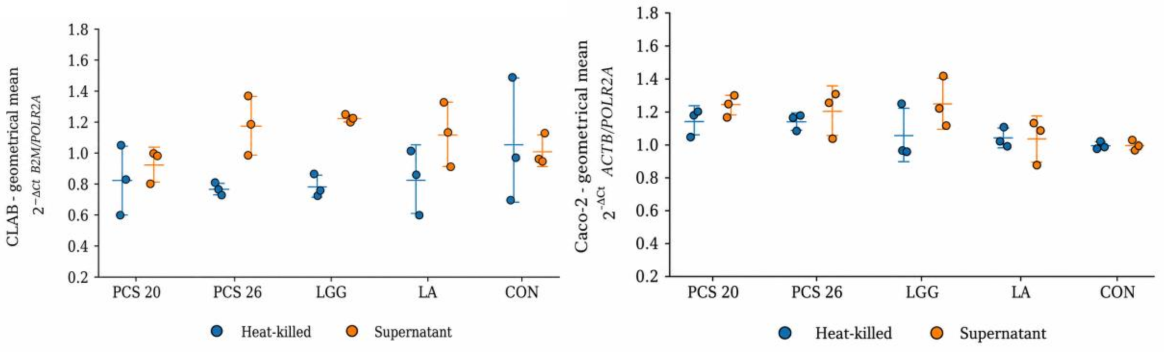

Figure S3. Expression of reference genes in CLAB (a) and Caco-2 (b) cells. Both cell lines were incubated for 6 hours with heat-killed probiotic strains and supernatants of the probiotic strains *Lactiplantibacillus plantarum* PCS 20 (PCS 20), *Lactiplantibacillus plantarum* PCS 26 (PCS 26), *Lacticaseibacillus rhamnosus* GG (LGG) and *Lactobacillus acidophilus* (LA). Data are presented as mean values $2^{-\Delta Ct}$ of geometric means of *B2M*/*POLR2A* (a) and *ACTB*/*POLR2A* (b) reference genes from three independent biological experiments (n = 3) with minimal and maximal expression error. Statistical differences between groups (*p>0.05) were determined by Anova and Welch test. CON: control; *B2M*: Beta-2-microglobulin; *POLR2A*: Polymerase II Subunit A; *ACTB*: Actin Beta.

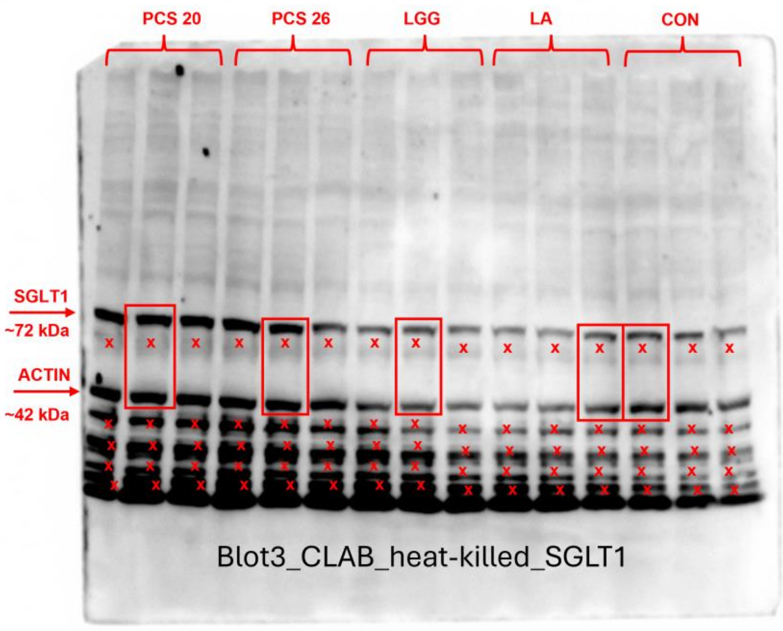

Figure S4. Immunoblot showing SGLT1 and ACTIN protein expression in CLAB cells after 6 h of incubation with heat-killed probiotic strains *Lactiplantibacillus plantarum* PCS 20 (PCS 20), *Lactiplantibacillus plantarum* PCS 26 (PCS 26), *Lacticaseibacillus rhamnosus* GG (LGG), and *Lactobacillus acidophilus* (LA), and the corresponding control (CON). SGLT1 and ACTIN were detected at approximately 72 kDa and 42 kDa, respectively. Three independent biological experiments (n = 3) are shown for each experimental condition. Bands not included in the final manuscript figure are marked with an "X". Rectangles indicate the SGLT1 and corresponding ACTIN bands selected as representative bands for presentation in Figure 4a of the manuscript. SGLT1: sodium-driven glucose transporter 1.

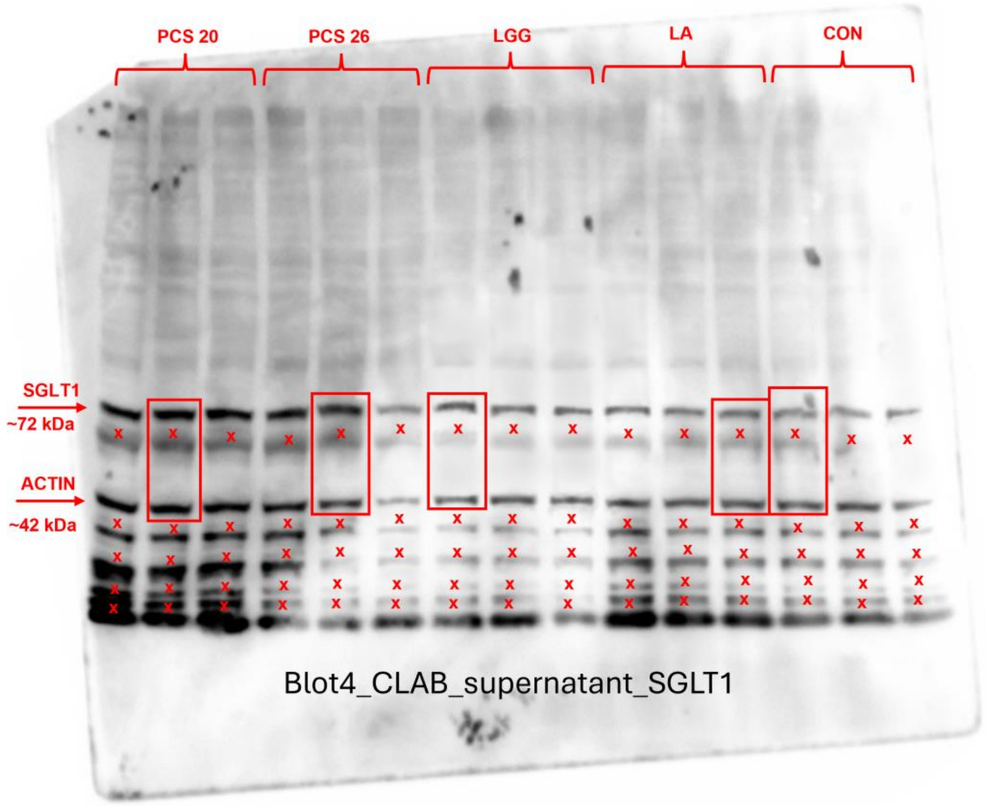

Figure S5. Immunoblot showing SGLT1 and ACTIN protein expression in CLAB cells after 6 h of incubation with cell-free supernatants of the probiotic strains *Lactiplantibacillus plantarum* PCS 20 (PCS 20), *Lactiplantibacillus plantarum* PCS 26 (PCS 26), *Lacticaseibacillus rhamnosus* GG (LGG), and *Lactobacillus acidophilus* (LA), and the corresponding control (CON). SGLT1 and ACTIN were detected at approximately 72 kDa and 42 kDa, respectively. Three independent biological experiments (n = 3) are shown for each experimental condition. Bands not included in the final manuscript figure are marked with an "X". Rectangles indicate the SGLT1 and corresponding ACTIN bands selected as representative bands for presentation in Figure 4a of the manuscript. SGLT1: sodium-driven glucose transporter 1.

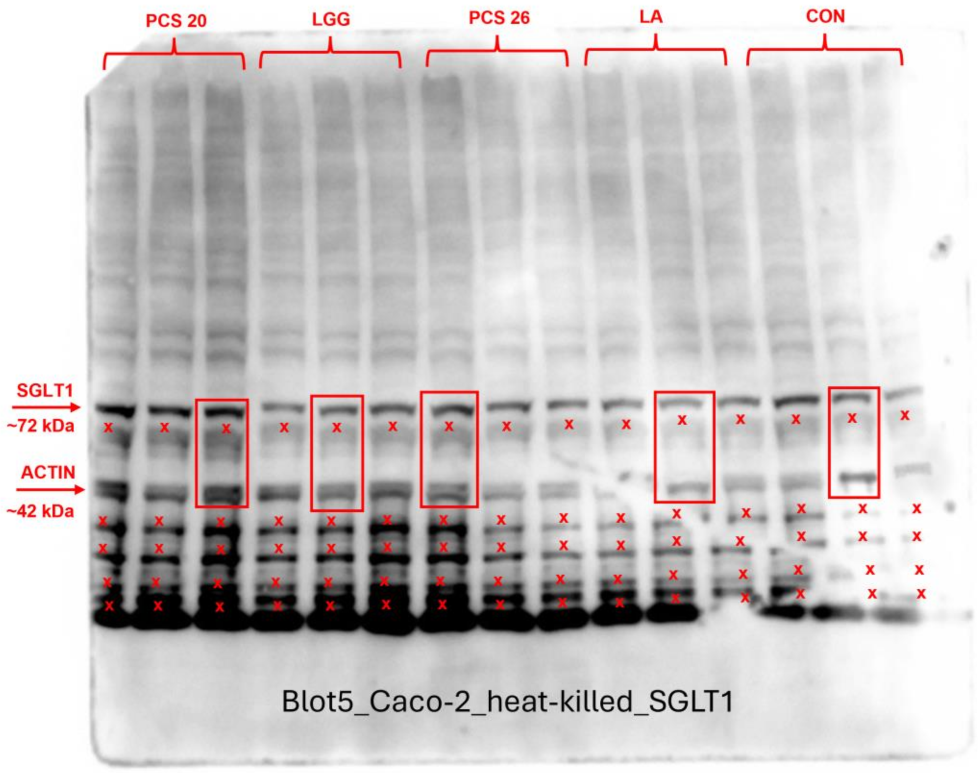

Figure S6. Immunoblot showing SGLT1 and ACTIN protein expression in Caco-2 cells after 6 h of incubation with heat-killed probiotic strains *Lactiplantibacillus plantarum* PCS 20 (PCS 20), *Lactiplantibacillus plantarum* PCS 26 (PCS 26), *Lacticaseibacillus rhamnosus* GG (LGG), and *Lactobacillus acidophilus* (LA), and the corresponding control (CON). SGLT1 and ACTIN were detected at approximately 72 kDa and 42 kDa, respectively. Three independent biological experiments (n = 3) are shown for each experimental condition. Bands not included in the final manuscript figure are marked with an "X". Rectangles indicate the SGLT1 and corresponding ACTIN bands selected as representative bands for presentation in Figure 4b of the manuscript. SGLT1: sodium-driven glucose transporter 1.

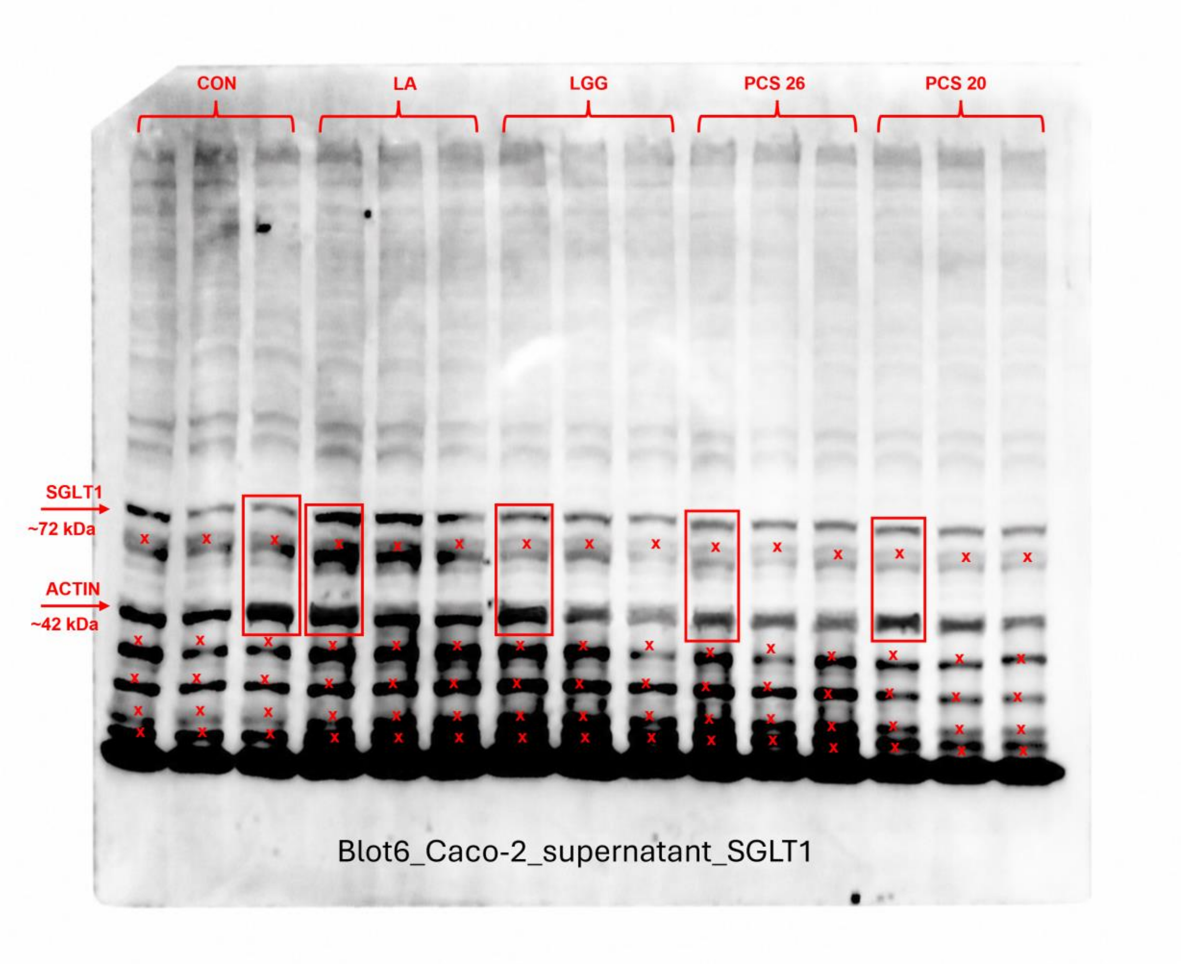

Figure S7. Immunoblot showing SGLT1 and ACTIN protein expression in Caco-2 cells after 6 h of incubation with cell-free supernatants of the probiotic strains *Lactiplantibacillus plantarum* PCS 20 (PCS 20), *Lactiplantibacillus plantarum* PCS 26 (PCS 26), *Lacticaseibacillus rhamnosus* GG (LGG), and *Lactobacillus acidophilus* (LA), and the corresponding control (CON). SGLT1 and ACTIN were detected at approximately 72 kDa and 42 kDa, respectively. Three independent biological experiments (n = 3) are shown for each experimental condition. Bands not included in the final manuscript figure are marked with an "X". Rectangles indicate the SGLT1 and corresponding ACTIN bands selected as representative bands for presentation in Figure 4b of the manuscript. SGLT1: sodium-driven glucose transporter 1.

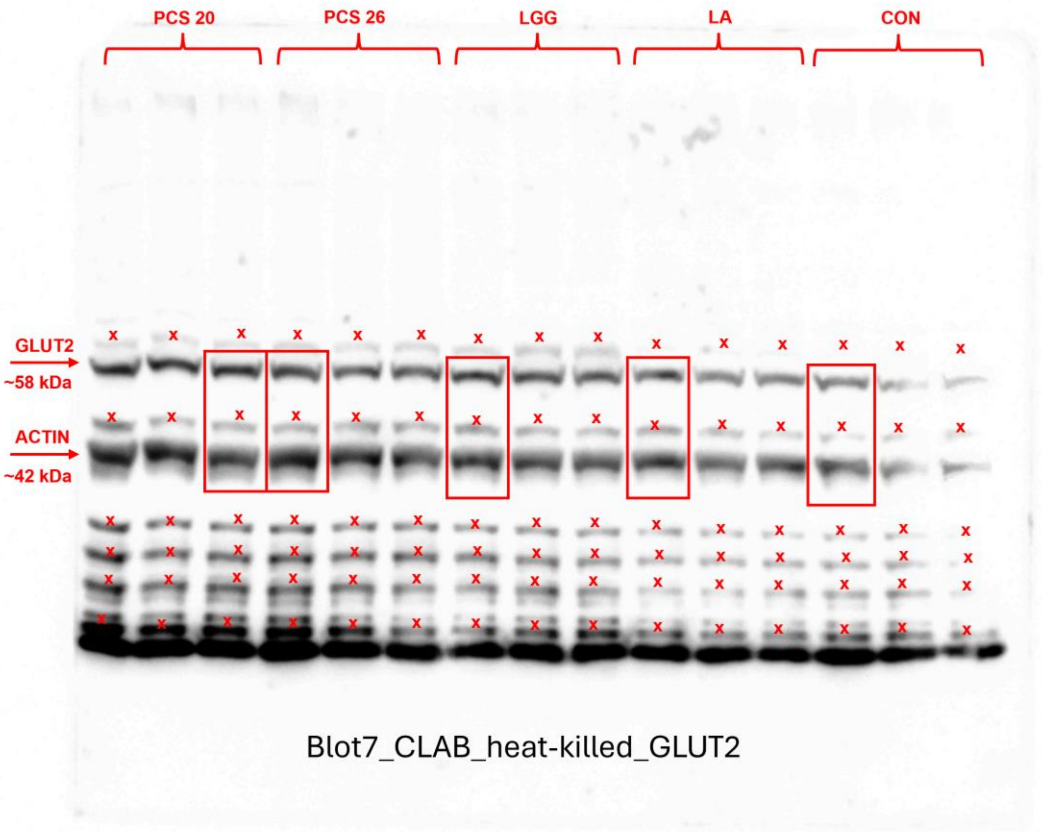

Figure S8. Immunoblot showing GLUT2 and ACTIN protein expression in CLAB cells after 6 h of incubation with heat-killed probiotic strains *Lactiplantibacillus plantarum* PCS 20 (PCS 20), *Lactiplantibacillus plantarum* PCS 26 (PCS 26), *Lacticaseibacillus rhamnosus* GG (LGG), and *Lactobacillus acidophilus* (LA), and the corresponding control (CON). GLUT2 and ACTIN were detected at approximately 58 kDa and 42 kDa, respectively. Three independent biological experiments (n = 3) are shown for each experimental condition. Bands not included in the final manuscript figure are marked with an "X". Rectangles indicate the GLUT2 and corresponding ACTIN bands selected as representative bands for presentation in Figure 5a of the manuscript. GLUT2: facilitative glucose transporter 2.

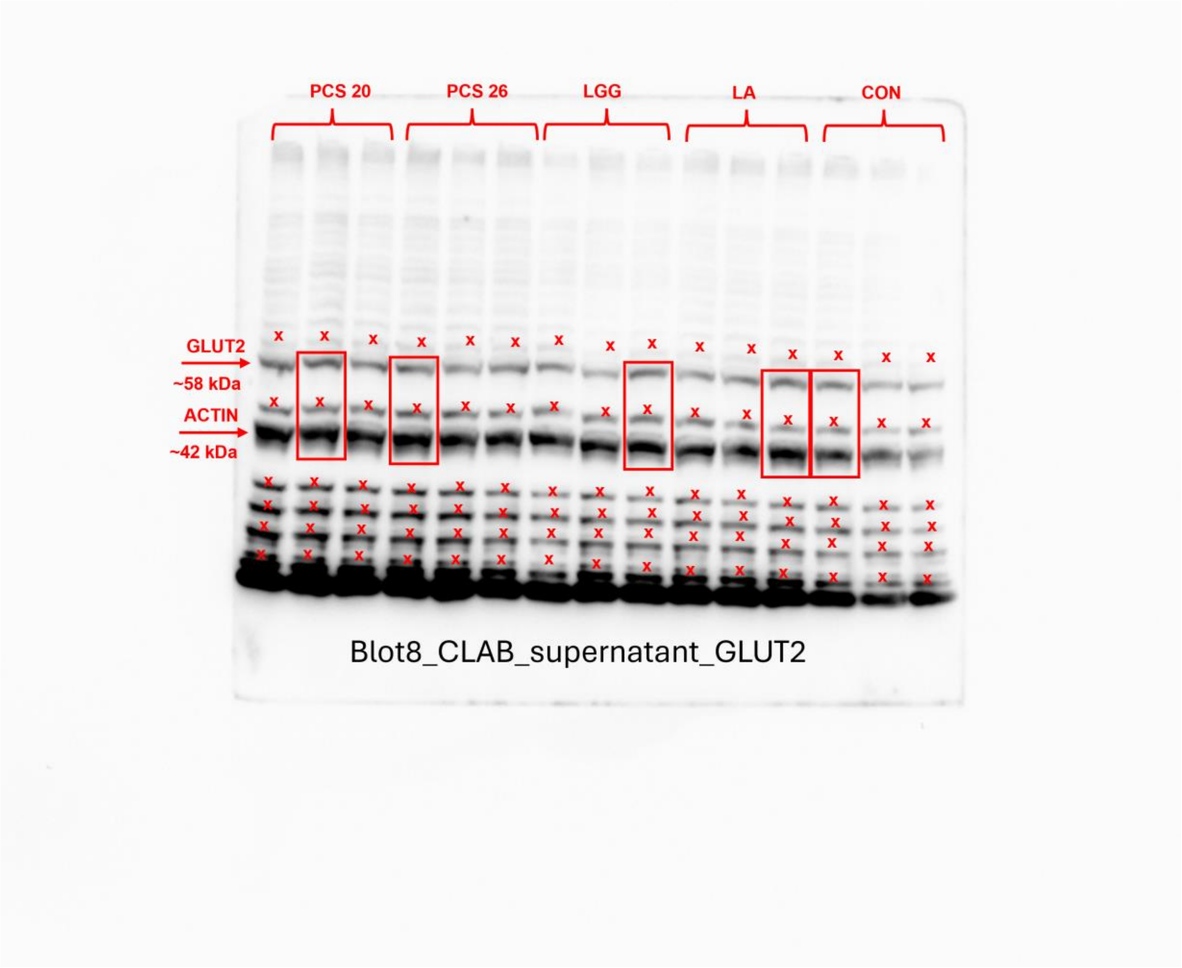

Figure S9. Immunoblot showing GLUT2 and ACTIN protein expression in CLAB cells after 6 h of incubation with cell-free supernatants of the probiotic strains *Lactiplantibacillus plantarum* PCS 20 (PCS 20), *Lactiplantibacillus plantarum* PCS 26 (PCS 26), *Lacticaseibacillus rhamnosus* GG (LGG), and *Lactobacillus acidophilus* (LA), and the corresponding control (CON). GLUT2 and ACTIN were detected at approximately 58 kDa and 42 kDa, respectively. Three independent biological experiments (n = 3) are shown for each experimental condition. Bands not included in the final manuscript figure are marked with an "X". Rectangles indicate the GLUT2 and corresponding ACTIN bands selected as representative bands for presentation in Figure 5a of the manuscript. GLUT2: facilitative glucose transporter 2.

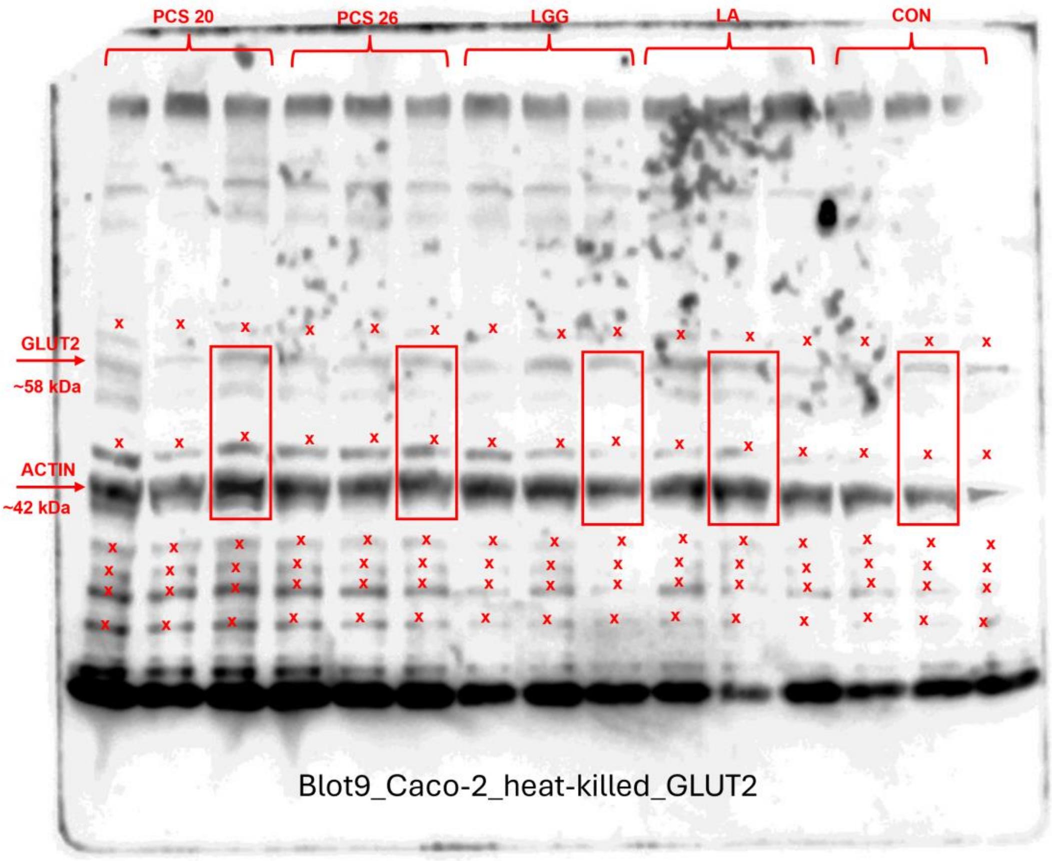

Figure S10. Immunoblot showing GLUT2 and ACTIN protein expression in Caco-2 cells after 6 h of incubation with heat-killed probiotic strains *Lactiplantibacillus plantarum* PCS 20 (PCS 20), *Lactiplantibacillus plantarum* PCS 26 (PCS 26), *Lacticaseibacillus rhamnosus* GG (LGG), and *Lactobacillus acidophilus* (LA), and the corresponding control (CON). GLUT2 and ACTIN were detected at approximately 58 kDa and 42 kDa, respectively. Three independent biological experiments (n = 3) are shown for each experimental condition. Bands not included in the final manuscript figure are marked with an "X". Rectangles indicate the GLUT2 and corresponding ACTIN bands selected as representative bands for presentation in Figure 5b of the manuscript. GLUT2: facilitative glucose transporter 2.

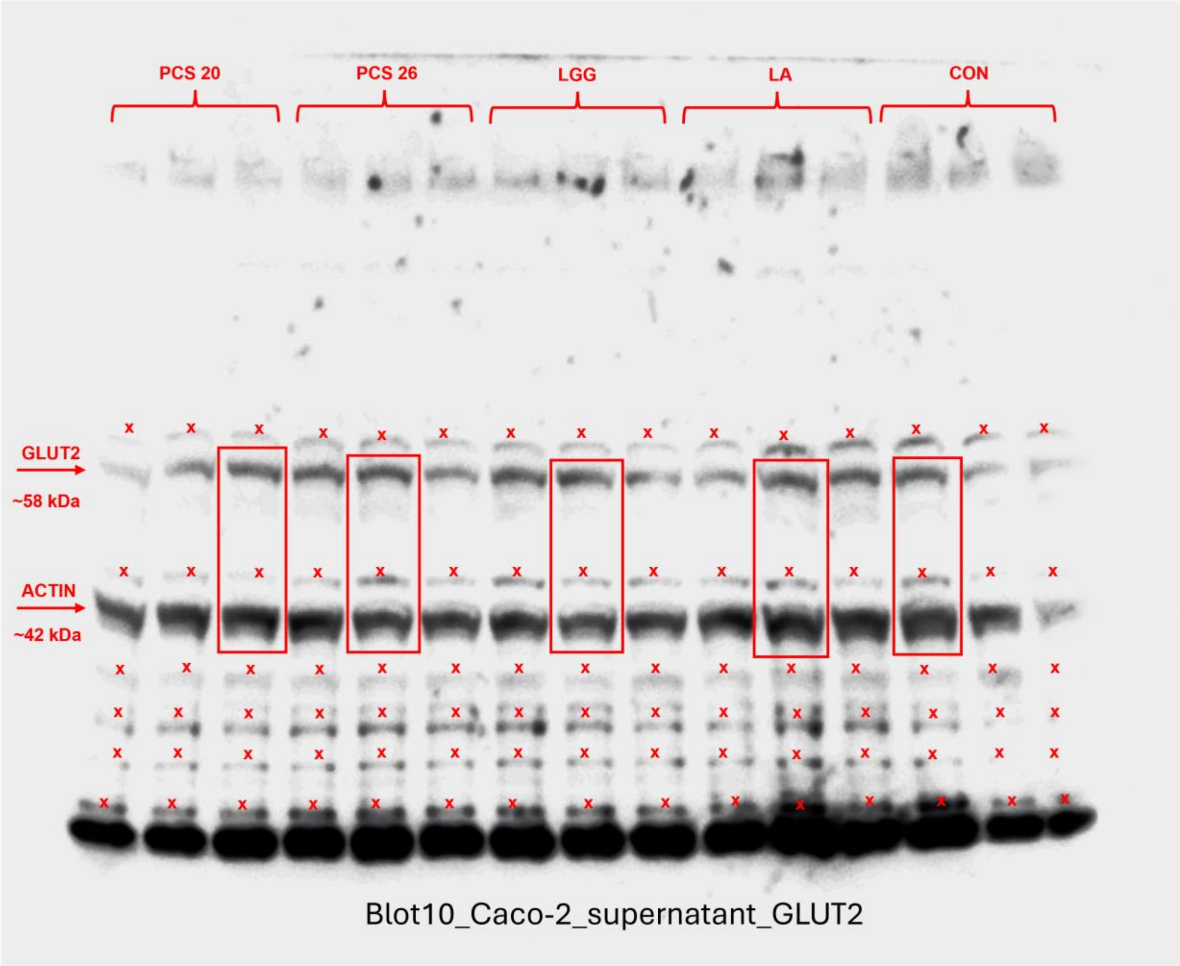

Figure S11. Immunoblot showing GLUT2 and ACTIN protein expression in Caco-2 cells after 6 h of incubation with cell-free supernatants of the probiotic strains *Lactiplantibacillus plantarum* PCS 20 (PCS 20), *Lactiplantibacillus plantarum* PCS 26 (PCS 26), *Lacticaseibacillus rhamnosus* GG (LGG), and *Lactobacillus acidophilus* (LA), and the corresponding control (CON). GLUT2 and ACTIN were detected at approximately 58 kDa and 42 kDa, respectively. Three independent biological experiments (n = 3) are shown for each experimental condition. Bands not included in the final manuscript figure are marked with an "X". Rectangles indicate the GLUT2 and corresponding ACTIN bands selected as representative bands for presentation in Figure 5b of the manuscript. GLUT2: facilitative glucose transporter 2.

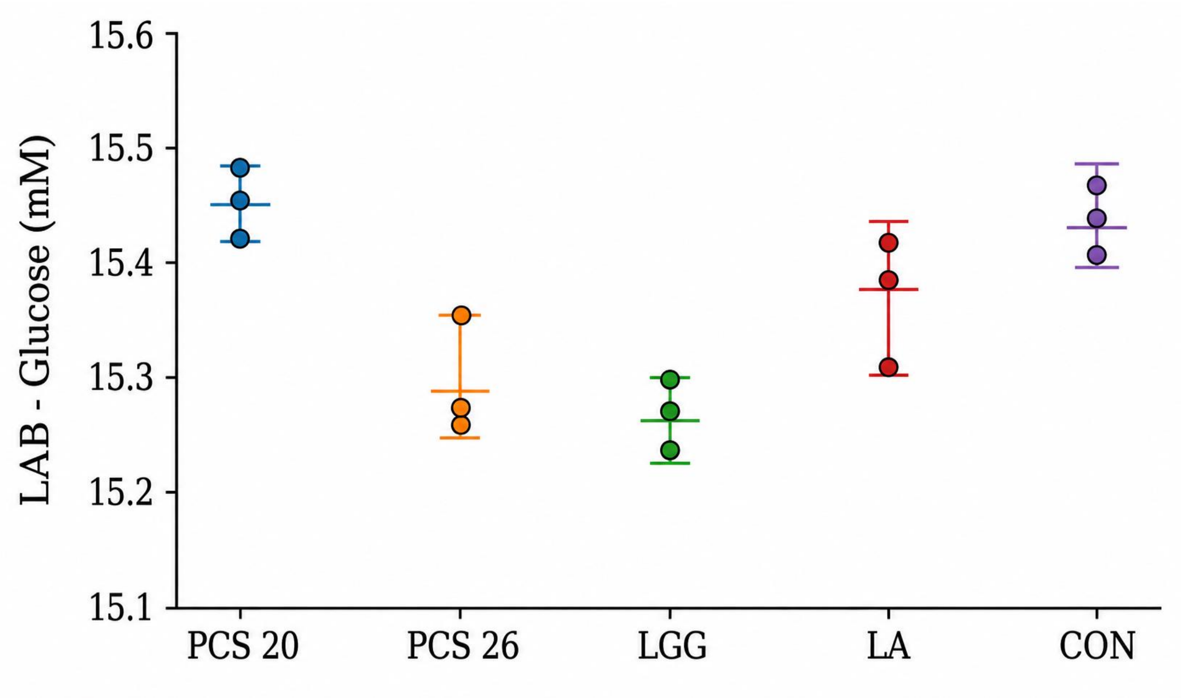

Figure S12. Glucose concentration measured after 6 h of incubation of the probiotic strains *Lactiplantibacillus plantarum* PCS 20 (PCS 20), *Lactiplantibacillus plantarum* PCS 26 (PCS 26), *Lacticaseibacillus rhamnosus* GG (LGG), and *Lactobacillus acidophilus* (LA) in DMEM cell culture medium. The initial concentration of each probiotic strain in DMEM was 1 × 10^7 CFU/mL. Data are presented as mean glucose concentrations ± SD from three independent biological experiments (n = 3), with individual data points shown. Significant differences compared with the corresponding probiotic-free control (CON) were assessed using the Kruskal–Wallis H-test (p < 0.05). No statistically significant differences were detected. LAB: Lactobacillaceae.

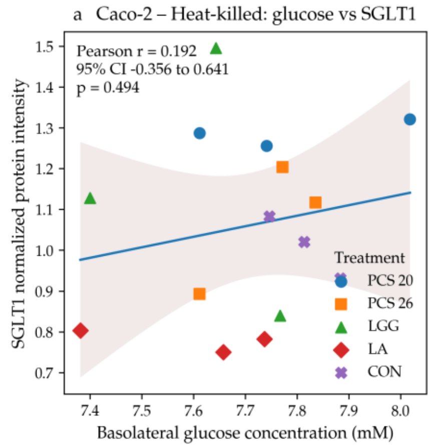
a Caco-2 – Heat-killed: glucose vs SGLT1
Pearson r = 0.192
95% CI -0.356 to 0.641
p = 0.494
Treatment
PCS 20
PCS 26
LGG
LA
CON
SGLT1 normalized protein intensity
Basolateral glucose concentration (mM)

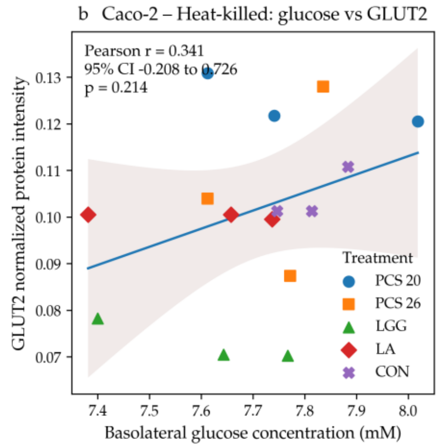
b Caco-2 – Heat-killed: glucose vs GLUT2
Pearson r = 0.341
95% CI -0.208 to 0.726
p = 0.214
Treatment
PCS 20
PCS 26
LGG
LA
CON
GLUT2 normalized protein intensity
Basolateral glucose concentration (mM)

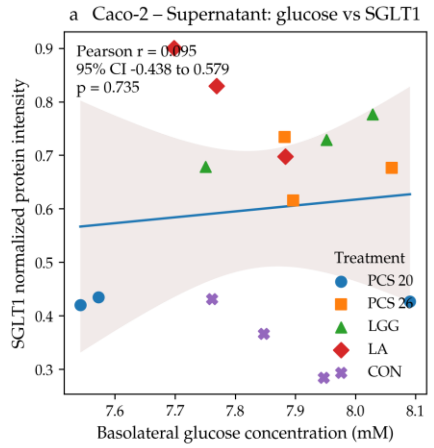
a Caco-2 – Supernatant: glucose vs SGLT1
Pearson r = 0.095
95% CI -0.438 to 0.579
p = 0.735
Treatment
PCS 20
PCS 26
LGG
LA
CON
SGLT1 normalized protein intensity
Basolateral glucose concentration (mM)

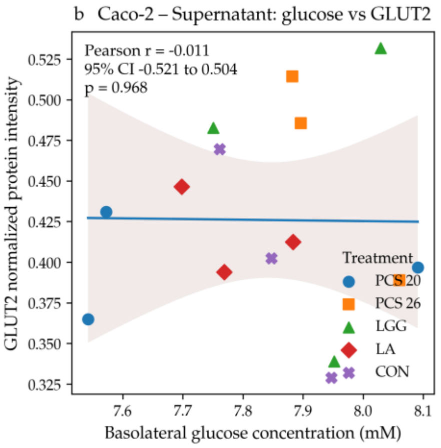
b Caco-2 – Supernatant: glucose vs GLUT2
Pearson r = -0.011
95% CI -0.521 to 0.504
p = 0.968
Treatment
PCS 20
PCS 26
LGG
LA
CON
GLUT2 normalized protein intensity
Basolateral glucose concentration (mM)

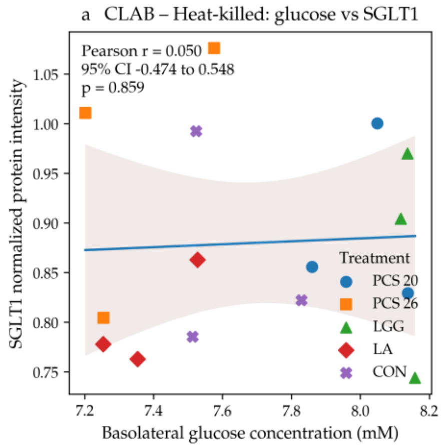
a CLAB – Heat-killed: glucose vs SGLT1
Pearson r = 0.050
95% CI -0.474 to 0.548
p = 0.859
Treatment
PCS 20
PCS 26
LGG
LA
CON
SGLT1 normalized protein intensity
Basolateral glucose concentration (mM)

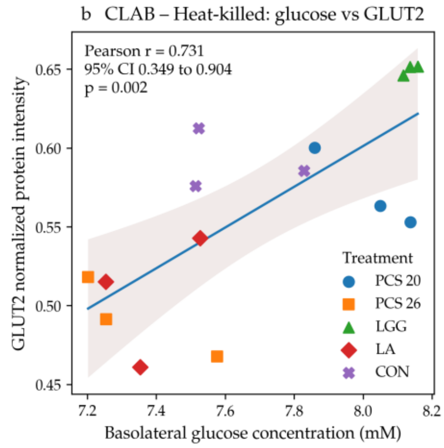
b CLAB – Heat-killed: glucose vs GLUT2
Pearson r = 0.731
95% CI 0.349 to 0.904
p = 0.002
Treatment
PCS 20
PCS 26
LGG
LA
CON
GLUT2 normalized protein intensity
Basolateral glucose concentration (mM)

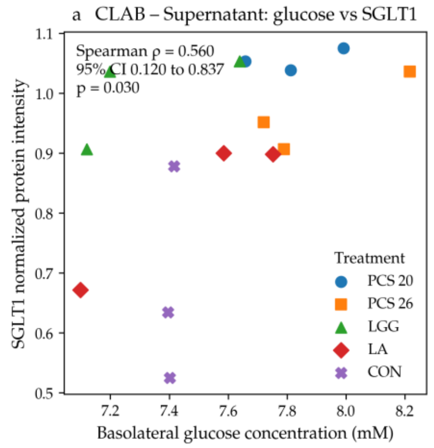
a CLAB – Supernatant: glucose vs SGLT1
Spearman ρ = 0.560
95% CI 0.120 to 0.837
p = 0.030
Treatment
PCS 20
PCS 26
LGG
LA
CON
SGLT1 normalized protein intensity
Basolateral glucose concentration (mM)

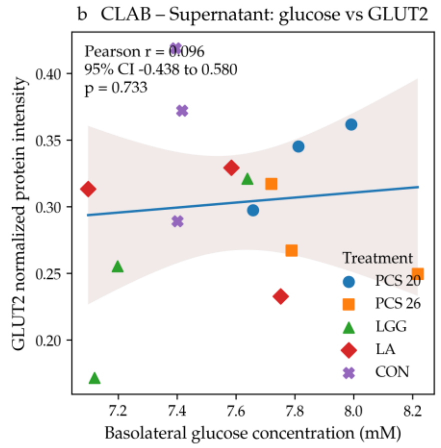
b CLAB – Supernatant: glucose vs GLUT2
Pearson r = 0.096
95% CI -0.438 to 0.580
p = 0.733
Treatment
PCS 20
PCS 26
LGG
LA
CON
GLUT2 normalized protein intensity
Basolateral glucose concentration (mM)

Figure S13. Correlation between basolateral glucose concentration and SGLT1 or GLUT2 protein expression in Caco-2 and CLAB cells following treatment with heat-killed probiotics or their cell-free supernatants. Scatterplots show individual paired biological observations for basolateral glucose concentration and normalised SGLT1 or GLUT2 protein intensity, with treatment identity indicated separately. Each treatment contributes $n = 3$ biological observations (total $n = 15$ per analysis). For analyses using Pearson's correlation coefficient (r), solid lines indicate the least-squares linear regression fit and shaded regions the 95% confidence interval of the fitted mean response. For the analysis using Spearman's rank correlation coefficient (ϱ), individual observations are shown without a fitted regression line. Correlation was assessed using Pearson's r or Spearman's ϱ, as appropriate. CI, confidence interval; CON: untreated control; PCS 20: *Lactiplantibacillus plantarum* PCS 20; PCS 26: *Lactiplantibacillus plantarum* PCS 26; LGG: *Lacticaseibacillus rhamnosus* GG; LA: *Lactobacillus acidophilus*.

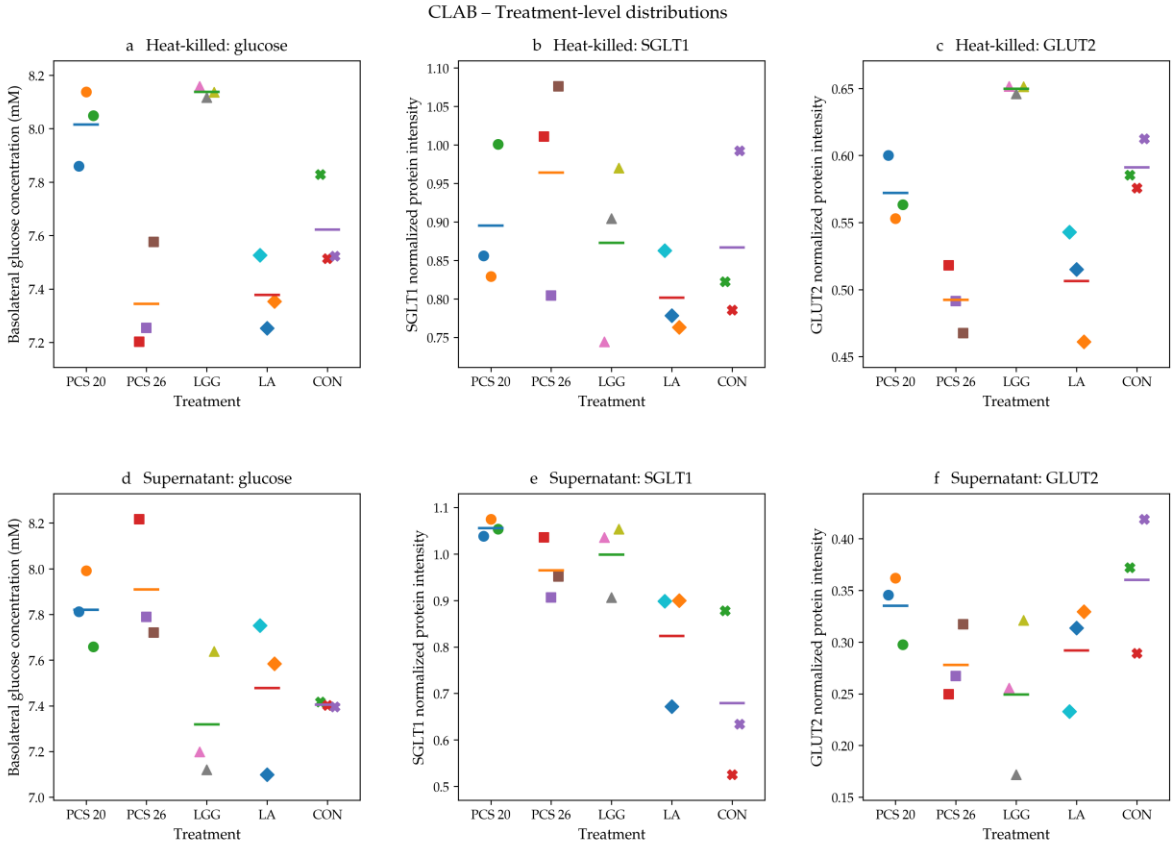

Figure S14. Treatment-level distribution of basolateral glucose concentration and SGLT1 and GLUT2 protein expression in CLAB cells following treatment with heat-killed probiotics or their cell-free supernatants. Individual symbols represent independent biological observations ($n = 3$ per treatment), and horizontal bars indicate the mean of the three biological observations. Panels (a–c) show basolateral glucose concentration, SGLT1 normalized protein intensity, and GLUT2 normalized protein intensity, respectively, following treatment with heat-killed probiotics. Panels (d–f) show the corresponding distributions following treatment with cell-free probiotic supernatants. CON: untreated control; PCS 20: *Lactiplantibacillus plantarum* PCS 20; PCS 26: *Lactiplantibacillus plantarum* PCS 26; LGG: *Lacticaseibacillus rhamnosus* GG; LA: *Lactobacillus acidophilus*.

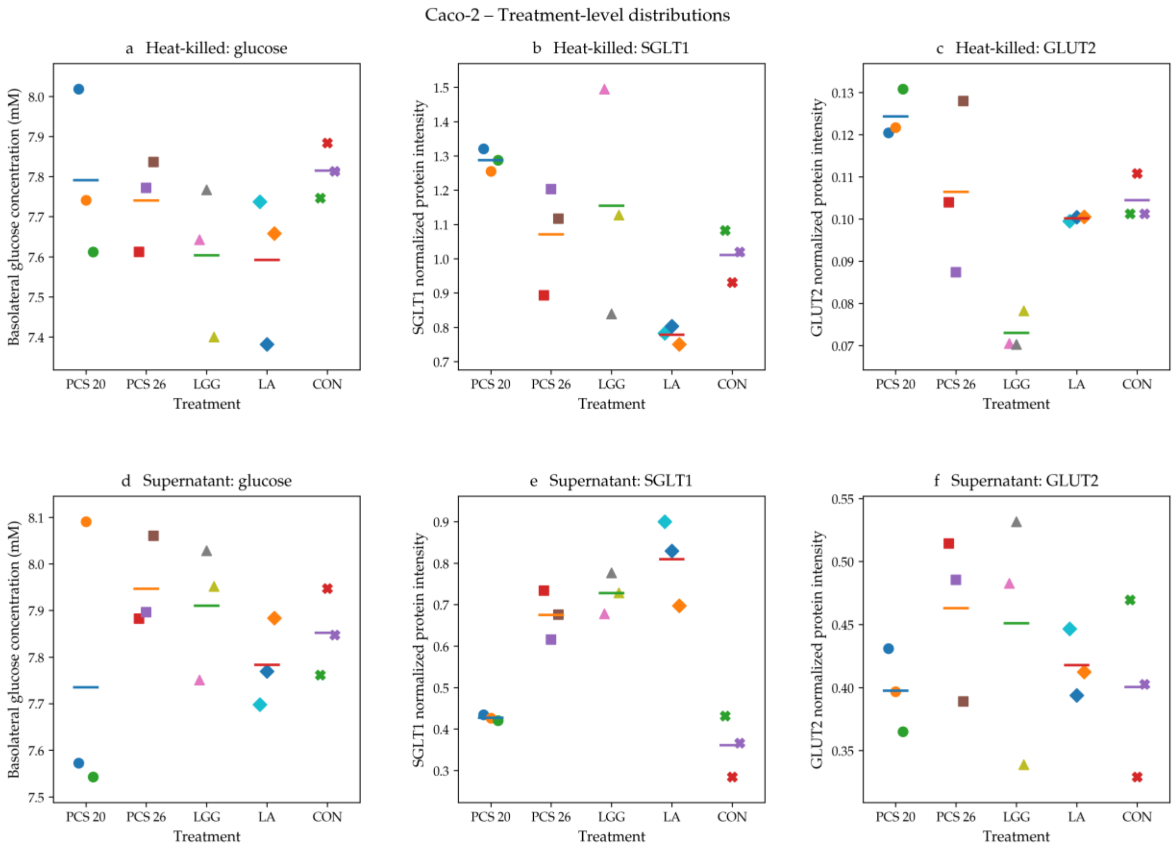

Figure S15. Treatment-level distribution of basolateral glucose concentration and SGLT1 and GLUT2 protein expression in Caco-2 cells following treatment with heat-killed probiotics or their cell-free supernatants. Individual symbols represent independent biological observations ($n = 3$ per treatment), and horizontal bars indicate the mean of the three biological observations. Panels (a–c) show basolateral glucose concentration, SGLT1 normalized protein intensity, and GLUT2 normalized protein intensity, respectively, following treatment with heat-killed probiotics. Panels (d–f) show the corresponding distributions following treatment with cell-free probiotic supernatants. CON: untreated control; PCS 20: *Lactiplantibacillus plantarum* PCS 20; PCS 26: *Lactiplantibacillus plantarum* PCS 26; LGG: *Lacticaseibacillus rhamnosus* GG; LA: *Lactobacillus acidophilus*.

Table S1. 6-h net basolateral glucose accumulation by probiotic treatment

Treatment		BL glucose 6h (mM)	Net BL glucose accumulation (µmol)	Area-normalized (µmol/cm²)	Apparent rate (µmol/cm²/h)
**CLAB**	**heat-killed**				
PCS 20*		8.016 ± 0.142	3.773 ± 0.213	3.773 ± 0.213	0.629 ± 0.035
PCS 26		7.344 ± 0.202	2.767 ± 0.304	2.767 ± 0.304	0.461 ± 0.051
LGG**		8.137 ± 0.021	3.956 ± 0.032	3.956 ± 0.032	0.659 ± 0.005
LA		7.378 ± 0.138	2.817 ± 0.207	2.817 ± 0.207	0.470 ± 0.035
Control		7.622 ± 0.179	3.183 ± 0.269	3.183 ± 0.269	0.530 ± 0.045
**Caco-2**	**heat-killed**				
PCS 20		7.791 ± 0.208	3.436 ± 0.311	3.436 ± 0.311	0.573 ± 0.052
PCS 26		7.740 ± 0.115	3.360 ± 0.173	3.360 ± 0.173	0.560 ± 0.029
LGG		7.603 ± 0.187	3.155 ± 0.280	3.155 ± 0.280	0.526 ± 0.047
LA		7.592 ± 0.187	3.138 ± 0.280	3.138 ± 0.280	0.523 ± 0.047
Control		7.815 ± 0.069	3.472 ± 0.103	3.472 ± 0.103	0.579 ± 0.017
**CLAB**	**supernatant**				
PCS 20		7.820 ± 0.167	3.481 ± 0.250	3.481 ± 0.250	0.580 ± 0.042
PCS 26		7.909 ± 0.269	3.613 ± 0.404	3.613 ± 0.404	0.602 ± 0.067

LGG		7.319 ± 0.280	2.729 ± 0.420	2.729 ± 0.420	0.455 ± 0.070
LA		7.478 ± 0.339	2.967 ± 0.509	2.967 ± 0.509	0.495 ± 0.085
Control		7.405 ± 0.011	2.857 ± 0.016	2.857 ± 0.016	0.476 ± 0.003
**Caco-2**	**supernatant**				
PCS 20		7.735 ± 0.308	3.353 ± 0.462	3.353 ± 0.462	0.559 ± 0.077
PCS 26		7.946 ± 0.099	3.669 ± 0.148	3.669 ± 0.148	0.612 ± 0.025
LGG		7.910 ± 0.143	3.616 ± 0.215	3.616 ± 0.215	0.603 ± 0.036
LA		7.784 ± 0.094	3.425 ± 0.140	3.425 ± 0.140	0.571 ± 0.023
Control		7.852 ± 0.093	3.528 ± 0.140	3.528 ± 0.140	0.588 ± 0.023

BL: basolateral; data are presented as mean ± SD (n = 3). Differences among probiotic treatments within each cell line and preparation compared with the control were analysed by one-way ANOVA followed by Dunnett's multiple-comparisons test after Levene's test confirmed homogeneity of variances (*p < 0.05, **p < 0.01).

Table S2. Individual paired biological observations used for correlation analyses between basolateral glucose concentration and SGLT1 or GLUT2 protein expression.

Cell line	Treatment preparation	Treatment	Biological experiment	Basolateral glucose (mM)	SGLT1 normalized protein intensity	GLUT2 normalized protein intensity
Caco-2	Heat-killed	PCS 20	I	8.018	1.321	0.120
Caco-2	Heat-killed	PCS 20	II	7.741	1.256	0.122
Caco-2	Heat-killed	PCS 20	III	7.612	1.287	0.131
Caco-2	Heat-killed	PCS 26	I	7.612	0.893	0.104
Caco-2	Heat-killed	PCS 26	II	7.772	1.203	0.087
Caco-2	Heat-killed	PCS 26	III	7.836	1.117	0.128
Caco-2	Heat-killed	LGG	I	7.643	1.495	0.070
Caco-2	Heat-killed	LGG	II	7.767	0.840	0.070
Caco-2	Heat-killed	LGG	III	7.400	1.128	0.078
Caco-2	Heat-killed	LA	I	7.737	0.782	0.100
Caco-2	Heat-killed	LA	II	7.382	0.803	0.100
Caco-2	Heat-killed	LA	III	7.658	0.750	0.101
Caco-2	Heat-killed	CON	I	7.746	1.083	0.101
Caco-2	Heat-killed	CON	II	7.884	0.931	0.111
Caco-2	Heat-killed	CON	III	7.814	1.020	0.101
Caco-2	Supernatant	PCS 20	I	7.572	0.434	0.431
Caco-2	Supernatant	PCS 20	II	8.091	0.427	0.397
Caco-2	Supernatant	PCS 20	III	7.543	0.420	0.365
Caco-2	Supernatant	PCS 26	I	7.882	0.734	0.514
Caco-2	Supernatant	PCS 26	II	7.896	0.616	0.485
Caco-2	Supernatant	PCS 26	III	8.060	0.676	0.389
Caco-2	Supernatant	LGG	I	7.751	0.678	0.483
Caco-2	Supernatant	LGG	II	8.029	0.777	0.532
Caco-2	Supernatant	LGG	III	7.952	0.729	0.339
Caco-2	Supernatant	LA	I	7.698	0.900	0.447
Caco-2	Supernatant	LA	II	7.769	0.830	0.394
Caco-2	Supernatant	LA	III	7.884	0.697	0.412
Caco-2	Supernatant	CON	I	7.761	0.431	0.470
Caco-2	Supernatant	CON	II	7.947	0.284	0.329
Caco-2	Supernatant	CON	III	7.847	0.366	0.403
CLAB	Heat-killed	PCS 20	I	7.860	0.856	0.600
CLAB	Heat-killed	PCS 20	II	8.138	0.829	0.553
CLAB	Heat-killed	PCS 20	III	8.049	1.001	0.563

Cell line	Treatment preparation	Treatment	Biological experiment	Basolateral glucose (mM)	SGLT1 normalized protein intensity	GLUT2 normalized protein intensity
CLAB	Heat-killed	PCS 26	I	7.202	1.011	0.518
CLAB	Heat-killed	PCS 26	II	7.255	0.804	0.491
CLAB	Heat-killed	PCS 26	III	7.576	1.076	0.468
CLAB	Heat-killed	LGG	I	8.159	0.744	0.652
CLAB	Heat-killed	LGG	II	8.117	0.904	0.646
CLAB	Heat-killed	LGG	III	8.137	0.970	0.651
CLAB	Heat-killed	LA	I	7.527	0.863	0.543
CLAB	Heat-killed	LA	II	7.254	0.778	0.515
CLAB	Heat-killed	LA	III	7.354	0.763	0.461
CLAB	Heat-killed	CON	I	7.829	0.822	0.585
CLAB	Heat-killed	CON	II	7.513	0.785	0.576
CLAB	Heat-killed	CON	III	7.523	0.993	0.612
CLAB	Supernatant	PCS 20	I	7.812	1.038	0.345
CLAB	Supernatant	PCS 20	II	7.991	1.075	0.362
CLAB	Supernatant	PCS 20	III	7.658	1.053	0.298
CLAB	Supernatant	PCS 26	I	8.217	1.036	0.250
CLAB	Supernatant	PCS 26	II	7.789	0.907	0.267
CLAB	Supernatant	PCS 26	III	7.720	0.951	0.317
CLAB	Supernatant	LGG	I	7.199	1.036	0.255
CLAB	Supernatant	LGG	II	7.120	0.907	0.172
CLAB	Supernatant	LGG	III	7.639	1.053	0.321
CLAB	Supernatant	LA	I	7.751	0.898	0.233
CLAB	Supernatant	LA	II	7.098	0.671	0.313
CLAB	Supernatant	LA	III	7.584	0.900	0.329
CLAB	Supernatant	CON	I	7.417	0.878	0.372
CLAB	Supernatant	CON	II	7.402	0.525	0.289
CLAB	Supernatant	CON	III	7.395	0.634	0.419

Table S3. Correlation analyses between basolateral glucose concentration and SGLT1 or GLUT2 protein expression.

Cell line	Preparation	Association	n	Correlation method	Correlation coefficient	95% CI	Exact *p*
Caco-2	Heat-killed	Glucose vs SGLT1	15	Pearson product-moment correlation	r = 0.192	-0.356 to 0.641	0.494
Caco-2	Heat-killed	Glucose vs GLUT2	15	Pearson product-moment correlation	r = 0.341	-0.208 to 0.726	0.214
Caco-2	Supernatant	Glucose vs SGLT1	15	Pearson product-moment correlation	r = 0.095	-0.438 to 0.579	0.735
Caco-2	Supernatant	Glucose vs GLUT2	15	Pearson product-moment correlation	r = -0.011	-0.521 to 0.504	0.968
CLAB	Heat-killed	Glucose vs SGLT1	15	Pearson product-moment correlation	r = 0.050	-0.474 to 0.548	0.859
CLAB	Heat-killed	Glucose vs GLUT2	15	Pearson product-moment correlation	r = 0.731	0.349 to 0.904	0.002
CLAB	Supernatant	Glucose vs SGLT1	15	Spearman rank correlation	rho = 0.560	0.120 to 0.837	0.030
CLAB	Supernatant	Glucose vs GLUT2	15	Pearson product-moment correlation	r = 0.096	-0.438 to 0.580	0.733

Each analysis included 15 paired treatment-level observations (n = 3 per treatment) obtained from three independent biological experiments. Pearson's product-moment correlation was used when normality assumptions were met; Spearman's rank correlation was used otherwise, based on the Shapiro-Wilk test. For Pearson's r, 95% confidence intervals (CI) were calculated using Fisher's z transformation; for Spearman's rho, the 95% CI was estimated using BCa bootstrap resampling (20,000 paired resamples; random seed 12345).

Table S4. Treatment-level distribution of basolateral glucose concentration and SGLT1 and GLUT2 protein expression.

Cell line	Preparation	Treatment	n	Glucose mean ± SD	Glucose min-max	SGLT1 mean ± SD	SGLT1 min-max	GLUT2 mean ± SD	GLUT2 min-max
Caco-2	Heat-killed	PCS 20	3	7.791 ± 0.208	7.612-8.018	1.288 ± 0.033	1.256-1.321	0.124 ± 0.006	0.120-0.131
Caco-2	Heat-killed	PCS 26	3	7.740 ± 0.115	7.612-7.836	1.071 ± 0.160	0.893-1.203	0.106 ± 0.020	0.087-0.128
Caco-2	Heat-killed	LGG	3	7.603 ± 0.187	7.400-7.767	1.154 ± 0.329	0.840-1.495	0.073 ± 0.005	0.070-0.078
Caco-2	Heat-killed	LA	3	7.592 ± 0.187	7.382-7.737	0.779 ± 0.027	0.750-0.803	0.100 ± 0.001	0.100-0.101
Caco-2	Heat-killed	CON	3	7.815 ± 0.069	7.746-7.884	1.011 ± 0.076	0.931-1.083	0.104 ± 0.006	0.101-0.111
Caco-2	Supernatant	PCS 20	3	7.735 ± 0.308	7.543-8.091	0.427 ± 0.007	0.420-0.434	0.398 ± 0.033	0.365-0.431
Caco-2	Supernatant	PCS 26	3	7.946 ± 0.099	7.882-8.060	0.675 ± 0.059	0.616-0.734	0.463 ± 0.066	0.389-0.514
Caco-2	Supernatant	LGG	3	7.910 ± 0.143	7.751-8.029	0.728 ± 0.049	0.678-0.777	0.451 ± 0.100	0.339-0.532
Caco-2	Supernatant	LA	3	7.784 ± 0.094	7.698-7.884	0.809 ± 0.103	0.697-0.900	0.418 ± 0.027	0.394-0.447
Caco-2	Supernatant	CON	3	7.852 ± 0.093	7.761-7.947	0.361 ± 0.074	0.284-0.431	0.400 ± 0.070	0.329-0.470
CLAB	Heat-killed	PCS 20	3	8.016 ± 0.142	7.860-8.138	0.895 ± 0.092	0.829-1.001	0.572 ± 0.025	0.553-0.600
CLAB	Heat-killed	PCS 26	3	7.344 ± 0.202	7.202-7.576	0.964 ± 0.142	0.804-1.076	0.492 ± 0.025	0.468-0.518
CLAB	Heat-killed	LGG	3	8.137 ± 0.021	8.117-8.159	0.873 ± 0.116	0.744-0.970	0.650 ± 0.003	0.646-0.652
CLAB	Heat-killed	LA	3	7.378 ± 0.138	7.254-7.527	0.801 ± 0.054	0.763-0.863	0.506 ± 0.042	0.461-0.543
CLAB	Heat-killed	CON	3	7.622 ± 0.179	7.513-7.829	0.867 ± 0.110	0.785-0.993	0.591 ± 0.019	0.576-0.612
CLAB	Supernatant	PCS 20	3	7.820 ± 0.167	7.658-7.991	1.056 ± 0.018	1.038-1.075	0.335 ± 0.033	0.298-0.362
CLAB	Supernatant	PCS 26	3	7.909 ± 0.269	7.720-8.217	0.965 ± 0.066	0.907-1.036	0.278 ± 0.035	0.250-0.317
CLAB	Supernatant	LGG	3	7.319 ± 0.280	7.120-7.639	0.999 ± 0.080	0.907-1.053	0.249 ± 0.075	0.172-0.321
CLAB	Supernatant	LA	3	7.478 ± 0.339	7.098-7.751	0.823 ± 0.132	0.671-0.900	0.292 ± 0.052	0.233-0.329
CLAB	Supernatant	CON	3	7.405 ± 0.011	7.395-7.417	0.679 ± 0.181	0.525-0.878	0.360 ± 0.066	0.289-0.419

Values are descriptive summaries of the three biological observations per treatment (n = 3). Basolateral glucose concentration is expressed in mM; SGLT1 and GLUT2 values represent normalized protein intensity. Data are presented as mean ± SD and minimum-maximum. These treatment-level summaries are provided to show the distribution of the observations and were not used as independent observations in the correlation analyses. CON: untreated control; PCS 20: *Lactiplantibacillus plantarum* PCS 20; PCS 26: *Lactiplantibacillus plantarum* PCS 26; LGG: *Lacticaseibacillus rhamnosus* GG; LA: *Lactobacillus acidophilus*.
